# Loss of the DNA Methyltransferase MET1 Induces H3K9 Hypermethylation at PcG Target Genes and Redistribution of H3K27 Trimethylation to Transposons in *Arabidopsis thaliana*

**DOI:** 10.1371/journal.pgen.1003062

**Published:** 2012-11-29

**Authors:** Angelique Deleris, Hume Stroud, Yana Bernatavichute, Elizabeth Johnson, Gregor Klein, Daniel Schubert, Steven E. Jacobsen

**Affiliations:** 1 Department of Molecular, Cell, and Developmental Biology, University of California Los Angeles, Los Angeles, California, United States of America; 2 Department of Biological Chemistry, David Geffen School of Medicine, University of California Los Angeles, Los Angeles, California, United States of America; 3 Institute of Genetics, Heinrich-Heine-University Düsseldorf, Düsseldorf, Germany; 4 Howard Hughes Medical Institute, University of California Los Angeles, Los Angeles, California, United States of America; University of Cambridge, United Kingdom

## Abstract

Dimethylation of histone H3 lysine 9 (H3K9m2) and trimethylation of histone H3 lysine 27 (H3K27m3) are two hallmarks of transcriptional repression in many organisms. In *Arabidopsis thaliana*, H3K27m3 is targeted by Polycomb Group (PcG) proteins and is associated with silent protein-coding genes, while H3K9m2 is correlated with DNA methylation and is associated with transposons and repetitive sequences. Recently, ectopic genic DNA methylation in the CHG context (where H is any base except G) has been observed in globally DNA hypomethylated mutants such as *met1*, but neither the nature of the hypermethylated loci nor the biological significance of this epigenetic phenomenon have been investigated. Here, we generated high-resolution, genome-wide maps of both H3K9m2 and H3K27m3 in wild-type and *met1* plants, which we integrated with transcriptional data, to explore the relationships between these two marks. We found that ectopic H3K9m2 observed in *met1* can be due to defects in IBM1-mediated H3K9m2 demethylation at some sites, but most importantly targets H3K27m3-marked genes, suggesting an interplay between these two silencing marks. Furthermore, H3K9m2/DNA-hypermethylation at these PcG targets in *met1* is coupled with a decrease in H3K27m3 marks, whereas CG/H3K9m2 hypomethylated transposons become ectopically H3K27m3 hypermethylated. Our results bear interesting similarities with cancer cells, which show global losses of DNA methylation but ectopic hypermethylation of genes previously marked by H3K27m3.

## Author Summary

In plants and animals, repetitive DNA sequences and transposable elements are marked with DNA methylation, which is associated with methylation on lysine 9 of histone 3 (H3K9) and silencing. On the other hand, protein-coding genes, in particular the ones involved in differentiation processes, are targeted by Polycomb Group (PcG) proteins, which results in trimethylation of H3K27—another hallmark of transcriptional repression. These two systems of silencing are thought to be independent, but in this study we reveal an interplay between them. In the model plant *Arabidopsis* we show that, in a globally DNA–hypomethylated mutant, H3K27m3 marks can now be found at repeats and transposons; this is associated with a decrease of H3K27m3 at PcG targets, with some of them becoming targets of DNA and H3K9 methylation. Our data suggest that H3K27m3 prevents ectopic DNA/H3K9 methylation at cryptic DNA methylation targets, which could provide a novel significance for this mark with regard to genome integrity. In addition, this study reveals interesting similarities with cancer cells, which show global losses of DNA methylation but ectopic hypermethylation of genes previously marked by H3K27m3, and suggests the potential of *Arabidopsis* as a system for understanding mammalian developmental and cancer biology.

## Introduction

Post-transcriptional modifications of histone tails—and combinations thereof—are thought to define specific chromatin structures and transcriptional states across eukaryotes [1], [2]. In both animals and plants, trimethylation of histone 3 lysine 27 (H3K27) and dimethylation of histone 3 lysine 9 (H3K9) (and/or trimethylation in metazoa) are two, generally alternative, hallmarks of transcriptional repression. In *Arabidopsis thaliana*, H3K27m3 is deposited by Polycomb group (PcG) proteins in euchromatic domains containing protein-coding genes—in particular, transcription factors and genes involved in developmental transitions [3], [4], [5]. H3K27m3 marks are largely non-overlapping with H3K9m2 and cytosine DNA methylation, which are targeted to repeated sequences throughout the genome and associated with silent, constitutive heterochromatin [4]. However, H3K27m3 was found to mark selected transposons and repeated sequences in some particular contexts when they are DNA hypomethylated such as in the *met1* mutants [6] or in endosperms [7].

In mammals DNA methylation is usually observed exclusively in the CG-dinucleotide context, while in *Arabidopsis thaliana* cytosines are methylated in every context. At least three DNA methyltransferases control DNA methylation in *Arabidopsis*, each with its own sequence preference: CG, CHG, or CHH (where H is any base except G). Establishment of cytosine methylation in all sequence contexts is catalyzed by DOMAINS REARRANGED METHYLTRANSFERASE 2 (DRM2), the plant homolog of mammalian DNMT3a and DNMT3b. DRM2 is guided to chromatin by siRNAs in a pathway known as RNA-directed DNA methylation (RdDM). This pathway also maintains DNA methylation in the asymmetric CHH context. CG methylation is catalyzed by DNA METHYLTRANSFERASE 1 (MET1), the plant homolog of mammalian DNMT1, and this mark is passively maintained during replication. Finally, CHG methylation is mostly catalyzed by CHROMOMETHYLASE 3 (CMT3)—a plant-specific DNA methyltransferase that contains a chromodomain that recognizes dimethylated histone tails at lysine 9 (H3K9m2) [8]. In turn, CHG methylation is recognized by the SRA domain within the KRYPTONITE (KYP) H3K9m2 methyltransferase. Therefore, CHG methylation is largely maintained through a reinforcing loop of DNA and H3K9 methylation. This is consistent with genome-wide studies showing that CHG methylation and H3K9m2 are highly co-incidental [9], [10]. At some heterochromatic loci, H3K9m2 is also dependent on CG methylation, potentially through other SRA-domain containing proteins [11], [12].

Primary targets of DNA methylation in *Arabidopsis* include transposons, repetitive sequences, and occasionally genes when they contain repeats in their promoter [13], [14]. In these cases, DNA methylation is present in all three cytosine contexts and is associated with H3K9m2 marks and transcriptional silencing [9], [15]. However, at least 30% of expressed genes [15], [16], [17], [18], [19] show a significant amount of DNA methylation in their transcribed regions (or gene bodies). In this case, DNA methylation is dependent on MET1, is restricted to CG sites, is not associated with H3K9m2, and does not result in gene silencing [17], [18]. The function of this methylation remains unknown, although a recent study gave some insights into its regulation: hundreds of genes in *Arabidopsis* were shown to gain non-CG methylation (mainly at CHG sites) in plants mutated in the *increase in bonsai methylation 1* (*ibm1*) gene [20]. *IBM1* encodes a Jumonji C-domain protein with H3K9m2 demethylase activity [21], [22], and was initially identified in a genetic screen for mutants showing ectopic cytosine methylation of the *BONSAI* (*BNS*) gene. This discovery raised the idea that genes are actively being protected from acquiring H3K9m2 methylation. Interestingly, ectopic CHG methylation and associated H3K9m2 have been previously reported in the *met1* background [16], [23], [24], [25]. However, neither the mechanism nor the biological significance of the ectopic DNA/H3K9m2 methylation in *met1* is currently understood.

In order to gain a better understanding of the ectopic DNA methylation in *met1*, and to test the hypothesis that CHG hypermethylation in *met1*, like in *ibm1*, could be the result of crippled IBM1-mediated control of H3K9m2 at genes, we generated high-resolution, genome-wide maps of H3K9m2 methylation in *met1* and *ibm1* mutants. Our results revealed that hundreds of genes become H3K9m2 hypermethylated in both *met1* and *ibm1* backgrounds. However, the sets of genes most H3K9m2 hypermethylated in each mutant were largely non-overlapping, suggesting that MET1 and IBM1 regulate H3K9m2 at different subsets of genes. The genes most H3K9m2 hypermethylated in *met1* tended to be either genes already marked with less extensive levels of H3K9m2 or, more surprisingly, genes marked with H3K27m3. To explore the relationship between the repressive H3K9m2 and H3K27m3 marks further, we mapped H3K27m3 levels using wild type and *met1* plants. We observed a significant loss of H3K27m3 at PcG-targeted genes in *met1*, in particular at the ones that became DNA and H3K9m2 hypermethylated in *met1*. This phenomenon was accompanied by a massive redistribution of H3K27m3 marks to many H3K9m2 and/or CG hypomethylated loci in *met1* such as transposons. Finally, to determine the effect of these changes in the epigenetic landscape on transcription, we conducted RNA-seq experiments using wild type and *met1* plants. These analyses showed that the PcG targets remain relatively unexpressed upon replacement of H3K27m3 marks by H3K9m2 marks. Our results suggest that H3K27m3 and H3K9m2/DNA methylation are mutually exclusive, and can replace one another in a locus specific manner. In addition, these data bring important new insight into the biology of *met1* mutants by showing an important role for MET1 in maintaining H3K27m3 patterning at PcG targets. Finally, our observations draw a striking parallel between the epigenetic phenomena displayed in the *met1* mutant and the local DNA hypermethylation observed in cancer cells.

## Results

### Genome-wide genic, differential H3K9m2 hypermethylation in *met1* and *ibm1* mutants

To examine the relationship between MET1 and IBM1 in negatively controlling H3K9m2 deposition throughout the genome, we generated high-resolution genome-wide maps of H3K9m2 in the two first inbred generations of *met1* and *ibm1* rosette-stage mutants by performing chromatin immunoprecipitation experiments coupled with whole-genome Roche Nimblegen microarray analyses (ChIP-chip). We observed hundreds of regions that became H3K9m2 hypermethylated in each of the mutants. However, while significant H3K9 hypermethylation was observed in the first generation of *ibm1* mutants (*ibm1*-1^st^), this phenomenon only become clearly apparent in the second generation of *met1* mutant (*met1*-2^nd^). These results are in contrast with previous immunofluorescence analyses showing appearance of H3K9m2 in the euchromatic, gene-rich regions only after three generations of the absence of a functional MET1 allele [25], but are consistent with genome-wide DNA methylation analyses where CHG ectopic methylation (and presumably H3K9 dimethylation) were evident in the flowers of *met1* first generation homozygous mutants [16]. This suggests that immunofluorescence experiments may not be sensitive enough to detect *de novo* H3K9m2 patterns in the second inbred generation.

**Figure 1:**
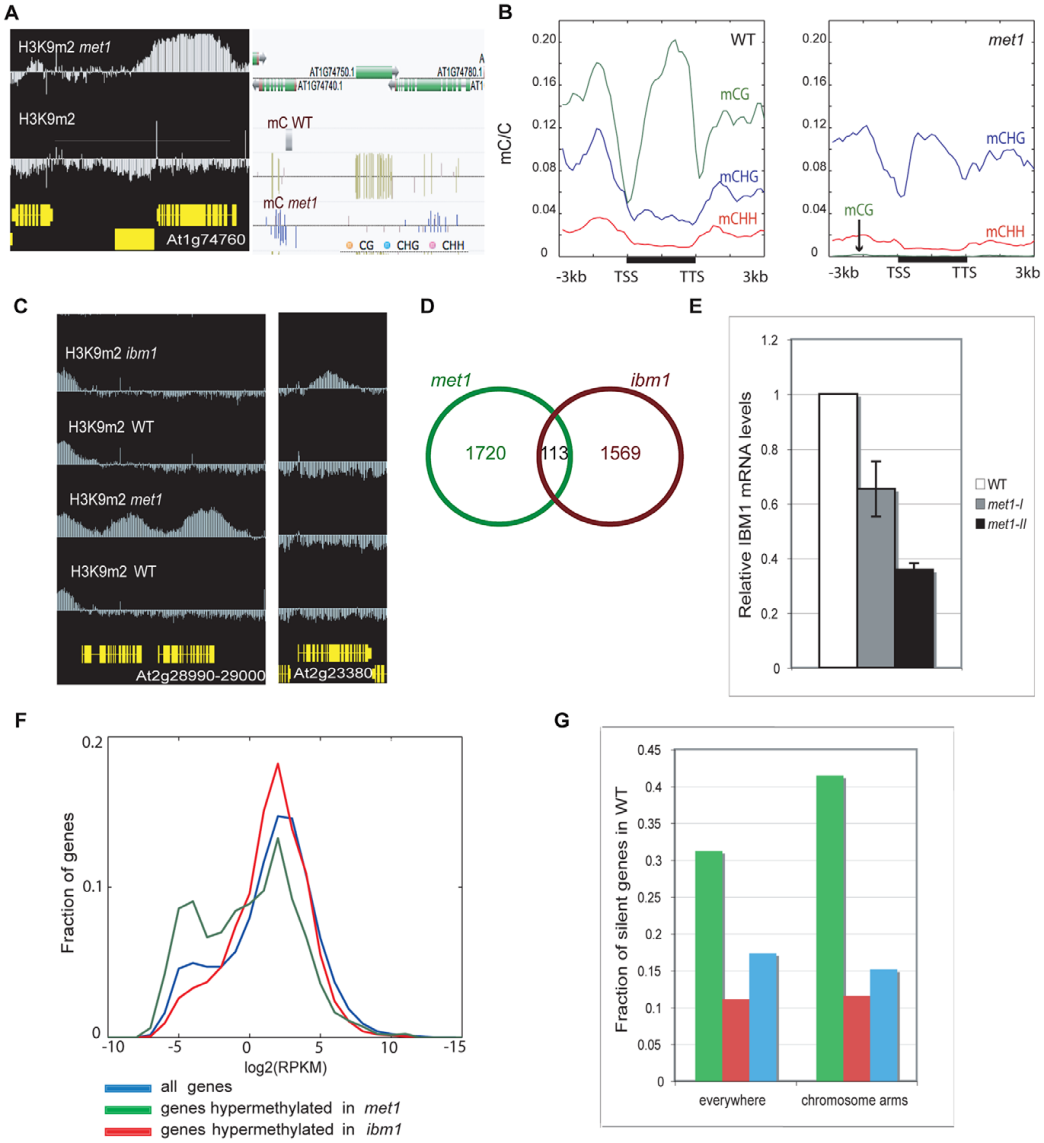
Genome-wide comparison of H3K9 hypermethylated genes in *met1* and *ibm1* mutants. A. Representative view of genes that gain ectopic H3K9m2 and non-CG methylation. Left panel: IGB (Integrative Genome Browser) view showing H3K9m2 levels. Yellow horizontal bars: protein-coding genes; vertical blue bar: relative H3K9m2 signal for each probe. Right panel: Anno-J browser view showing DNA methylation (methylC-seq) data [16] B. Distribution of DNA methylation across protein-coding genes that gain ectopic H3K9m2 in *met1*, in wild-type (left) and *met1* (right). The ratios of methylated cytosines to all cytosines were plotted over the genes (horizontal black bars) along with 3 kb upstream and downstream regions in 150 bp bins. Plots were smoothed by taking the moving average. TSS = transcription start site. TTS = transcription termination site. C. Left panel: representative view of two genes that gain H3K9m2 marks in their coding-region in *met1* but not in *ibm1*. Right panel: representative view of a gene that gains H3K9m2 marks in its body in *ibm1* but not in *met1*. D. Overlap of genes H3K9m2 hypermethylated in *met1* and *ibm1* mutants. E. IBM1 mRNA levels in *met1* backgrounds (*met1*-I: first generation; *met1*-II: second generation). mRNA was quantified by quantitative RT-PCR and normalized to an internal control (actin) and then to the wild type value. F. Expression levels of genes H3K9m2 hypermethylated in *ibm1* and *met1* compared to all genes. X axis = expression levels (reads per kilobase per million mapped reads (RPKM), log2 scale). Y axis = fraction of genes with the given expression level. G. Fraction of H3K9m2 hypermethylated genes that are silenced (No RNA-seq reads) in wild type, in the whole genome (left) and in chromosomal arms only (right). The color code is the same as in F.

Regions of H3K9m2 hypermethylation were defined (using the BLOC algorithm [26]) and all the analyses performed in the first generation for *ibm1* and the second generation for *met1*. The most hypermethylated genes were defined as genes that overlap with defined hypermethylated regions by at least 150 bases (the approximate length of DNA wrapped around one nucleosome). By this method, 1833 genes (6.49% of all genes, Table S1) were found to be H3K9m2 hypermethylated in *met1*. This set of genes included *SUPERMAN* (*SUP*) and *AGAMOUS* (*AG*), which have previously been reported to be DNA hypermethylated in *met1* [23], [24], [27] (Figure S1). In addition, we examined published whole genome bisulfite sequencing data obtained from flowers of first generation *met1* homozygous mutants, in which genic hypermethylation was readily detected [16]. We found that the set of genes that are H3K9 hypermethylated in *met1* in our experiment also displayed increased levels of non-CG methylation, particularly in the CHG context, at individual loci and in a genome-wide manner (Figure 1A, Figure 1B, Figure S1). This indicates that the feed-forward loop between H3K9 and CHG methylation is also active at ectopically methylated loci in *met1*. However, we note that the comparison between the patterns of DNA and H3K9 methylation is limited by the use of different developmental stages in the two studies.

In *ibm1* mutants, 1682 genes (5.96% of all genes, Table S2) were found to be H3K9m2-hypermethylated. Interestingly, this set of genes was largely distinct from the set observed for *met1*, with only 113 genes being significantly hypermethylated in both backgrounds (Figure 1C, 1D). These results imply that there are at least two mechanisms at play in the protection of genes from ectopic DNA and H3K9m2 methylation, one that depends on IBM1 and another that depends on MET1. One possibility is that the small overlap between the two sets is due to the reduced IBM1 mRNA levels in *met1* mutant (Figure 1E). Consistent with this notion, we found that *met1* usually had a smaller effect on H3K9m2 hypermethylation at these sites than *ibm1* (Figure S2). A recent study reported that the re-establishment of IBM1 expression in *met1* mutants restored the wild-type H3K9m2 patterns at selected loci and suggested that down-regulation of IBM1 could account for most of H3K9m2 relocation at genes [28]. However, the use of stringent thresholds to define H3K9m2 hypermethylated regions revealed that the most hypermethylated genes in *met1* are usually not targets of IBM1.

One difference between the characteristics of genes most hypermethylated in *ibm1* versus *met1* is that most of the H3K9m2 hypermethylated genes in *ibm1* are moderately expressed in wild-type, while many of the hypermethylated genes in *met1* are lowly expressed (Figure 1F) or silent (Figure 1G).

### Genes H3K9m2 hypermethylated in *met1* are pre-marked with either H3K9m2 methylation (Class I) or H3K27m3 methylation (Class II) and tend to be paralogous

**Figure 2:**
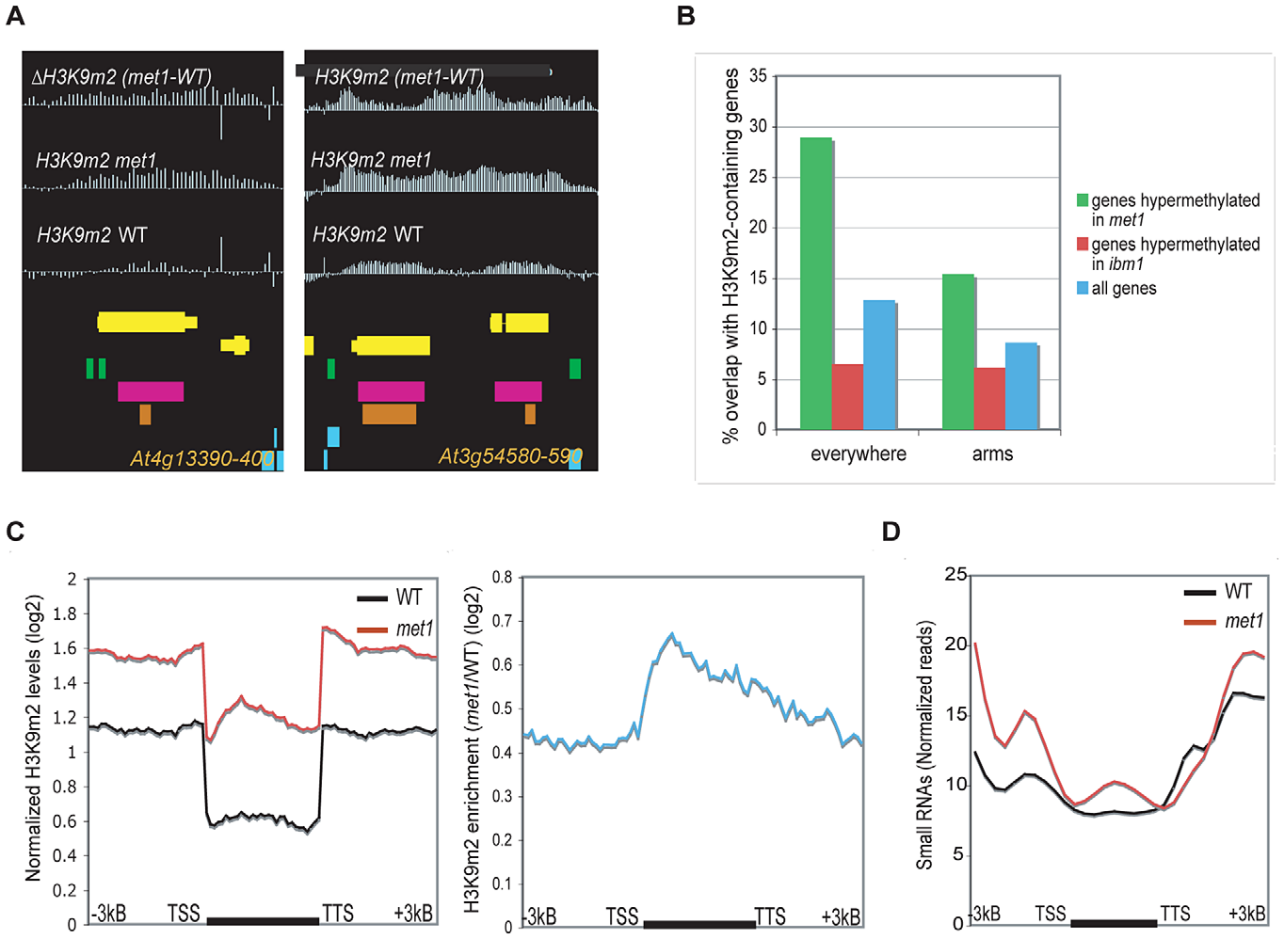
Genes that gain ectopic H3K9m2 in *met1* and are marked with H3K9m2 (Class I). A. Representative view of genes that gain H3K9m2 marks in their coding-region in *met1* and are pre-marked with H3K9m2 in wild type (Class I). Yellow horizontal bars: protein-coding genes; blue horizontal bars: transposable elements, green bars: dispersed repeats; purple bars: tandem repeats; orange bars: MPSS small RNA clusters. B. Fraction of genes that overlap with defined H3K9m2 regions in wild-type, in the whole genome (left) and in the chromosome arms only (right). C. Left panel: Average distributions of normalized H3K9m2 signals in wild-type and *met1* across protein-coding genes that display H3K9m2 in wild type and gain H3K9m2 in *met1* (Class I genes). Right panel: log2 *met1*/WT ratio of average H3K9m2 normalized values at Class I genes. TSS: transcription start site. TTS: transcription termination site. D. Distribution of small RNA-seq reads [16] across Class I genes.

By examination of individual loci (Figure 2A, Figure S3A) and genome-wide analysis (Figure 2B and 2C), we observed that many hypermethylated genes in *met1* were enriched for genes pre-marked with H3K9m2 in wild-type (“Class I” genes), presumably due to the presence of repeats within their coding-region (Figure 2A) that do not necessarily correspond to transposable elements (Figure S3B). 531 such “Class I” genes were identified (28.9% of all H3K9m2 hypermethylated genes in *met1*). At these genes, siRNAs levels were increased in *met1* (Figure 2D, Figure S3C), indicating that loss of CG DNA methylation at these genes, either upstream or in the coding-region, results in the stimulation of *de novo* methylation by the RdDM pathway, which in turn likely leads to increased H3K9m2 levels via the maintenance of CHG methylation which involves the H3K9m2 HMTase KRYPTONITE (KYP).

**Figure 3:**
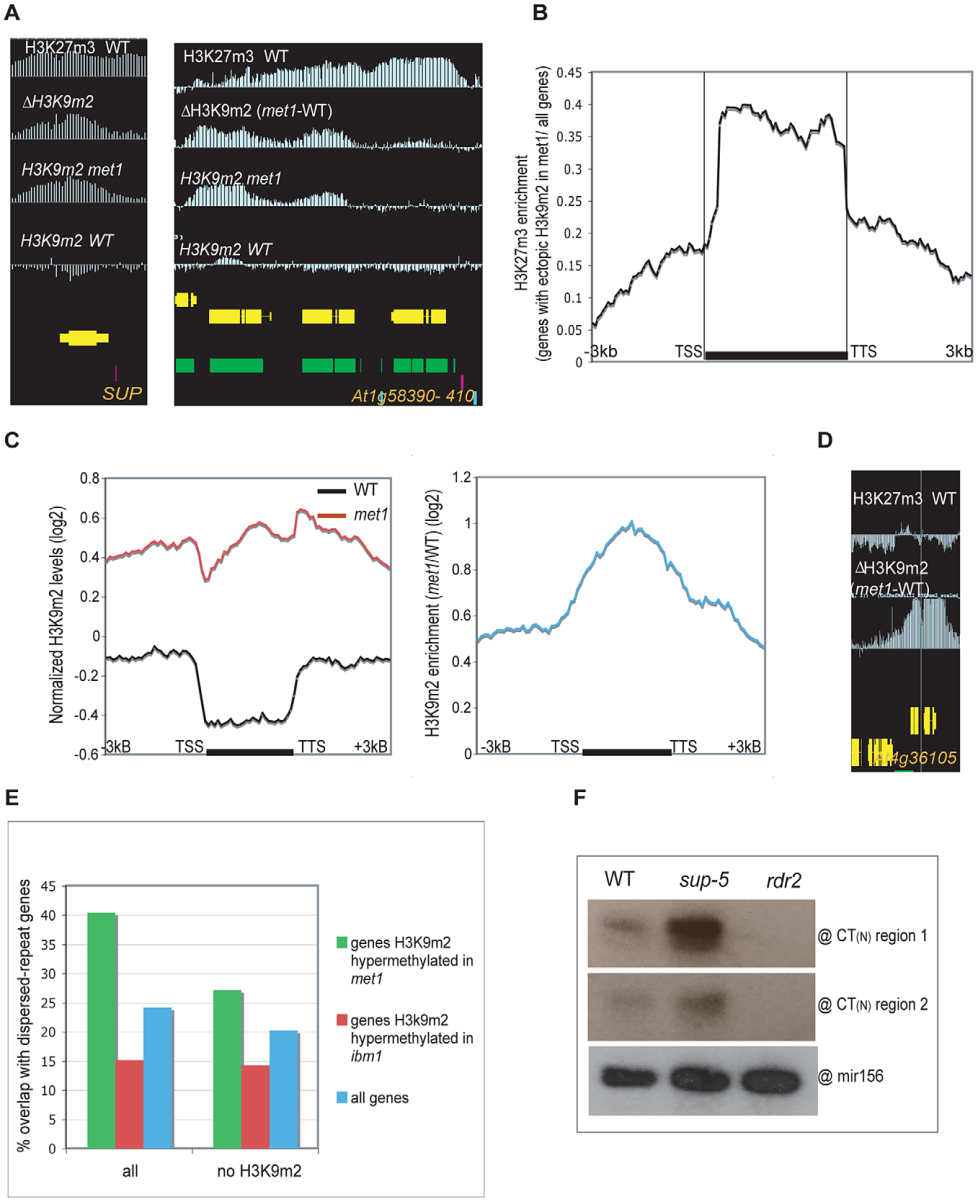
Genes that gain ectopic H3K9m2 in *met1* and are marked with H3K27m3 in wild-type (Class II). A. Representative view of genes that gain H3K9m2 marks in their coding-region in *met1* and are pre-marked with H3K27m3 in wild-type (Class II). B. Enrichment in H3K27m3 across protein-coding genes (horizontal black bar) that gain ectopic H3K9m2 in *met1*. This enrichment corresponds to the log2 ratio between the normalized H3K27m3 signals averaged across genes that gain ectopic H3K9m2 in *met1* and the normalized H3K27m3 signals averaged across all genes. C. Left panel: Average distribution of the normalized H3K9m2 signals in wild-type and *met1* across protein-coding genes that display H3K27m3 in wild type and gain H3K9m2 in *met1* (Class II genes). Right panel: log2 *met1*/WT ratio of average H3K9m2 normalized values at Class I genes. TSS: transcription start site. TTS: transcription termination site. D. Representative views of genes that gain H3K9m2 in *met1* and are H3K27m3-marked in wild type; however, these genes, due to their low levels of H3K27m3 in wild-type, were not recovered among Class II genes. This shows that the number of true Class II genes (H3K27m3 in wild type and ectopic H3K9m2 in *met1*) is likely underestimated by our stringencies. E. Fraction of genes that contain dispersed repeats (as annotated by [16]) in wild-type. Right: Fraction of genes that contain dispersed repeats but do not contain a H3K9m2 region (as defined in [9]) in wild-type. Notably, even though 40% of genes hypermethylated in *met1* contain dispersed repeats, a much larger proportion of them likely have regions of homology that were not annotated as dispersed repeats (due to the high stringency applied to define repeats). This is indeed the case for the *SUP* and *SUP-*like genes that show significant sequence similarity (At3g23130 and At2g42410, E-value 8e-26, 23% identity) and Citrate Synthase 1 and 2 (At3g58740 and At3g58750, E-value 1e-54, 54,3% identity) (shown in Figure S5C). F. Small RNA analysis by Northern Blot. The existence of SUP-hybridizing siRNAs (24–27 nt) in wild-type plants was confirmed by 2 probes corresponding to the first and second CT region respectively. These probes were also able to detect small RNAs in *sup-5* SUPERMAN deletion allele, which is devoid of the *SUP* coding-sequence. A probe detecting mir156 was used as a control.

We also observed a second class of H3K9m2 hypermethylated genes in *met1*, including *SUPERMAN* or *AGAMOUS*, which consistently display H3K27m3 marks in wild type plants at this same developmental stage (“Class II” genes) (Figure 3A and 3C, Figure S4A and S4B). With our stringent parameters, this class comprised 515 genes (28.1% of all H3K9m2 hypermethylated genes). However, this number is an under-estimation as we noted a large number of genes that had the characteristics of Class II genes but were not retrieved by our conservative cutoffs for defining H3K27m3-marked genes in wild type (Figure 3D, Figure S5A). These genes seemed to largely account for the H3K9m2 hypermethylated genes in *met1* that were not included in Class I or II (data not shown). In addition, genome-wide analyses revealed that genes H3K9m2 hypermethylated in *met1* are significantly enriched in H3K27m3 marks relative to all genes (Figure 3B). Together, these observations indicate that the ectopic H3K9m2/DNA methylation in *met1* is preferentially targeted to regions enriched in H3K27m3 in wild type. These observations are strikingly reminiscent of the phenomena displayed by human cancers cells where ectopic *de nov*o DNA methylation occurs predominantly at genes specifically marked with H3K27m3 in either the corresponding normal adult cells or in the progenitor cells [29], [30], [31].

While regions that gained H3K9m2 in *met1* were enriched for sites marked with H3K27m3 in wild type, only 7.8% of PcG-targeted genes (515 genes out of 6592) gain H3K9m2 in *met1* (Figure S5B). While this number is likely an under-estimation (due to our conservative cutoffs), this nonetheless indicates that additional factors must contribute to the observed phenomenon. To identify such factors, we looked for additional features of H3K9m2 hypermethylated genes in the mutant background and found that they were enriched in sequences annotated as “dispersed repeats”, but were not annotated as transposable elements but rather as regions of homology between gene families (Figure 3E, Figure S4). Specifically, genes H3K9m2 hypermethylated in *met1* were often found in tandem and had similar gene ontologies indicating recent gene duplication. In other cases, some H3K9m2 hypermethylated genes with paralogous domains were located on different chromosomes such as the transcriptions factors (*At3g58780*, *At2g42830*) related to *AGAMOUS (At4g18960)* by their MADS-box domain. Interestingly, tandemly repeated genes were also shown to be represented among H3K27m3-marked genes [3]. The homologous nature of the genes H3K9m2 hypermethylated in *met1* suggests that a sequence-specific process—such as RdDM—may be involved in the formation of these ectopic methylation patterns. The presence of *SUP* DNA hypermethylated alleles (also known as *clark kent* or *clk* alleles) in a globally hypomethylated background was previously shown to depend on both the CMT3 and RdDM pathway [32]. Interestingly, in wild-type plants, we detected *SUP*-hybridizing small interfering RNAs, that did not originate from the *SUP* locus (since they were still detected in a strain with a deletion of the *SUP* gene) (Figure 3F). This shows that *SUP*-hybridizing siRNAs produced by another locus might potentially target *SUP* in trans. Consistent with this idea, the gene families H3K9m2 hypermethylated in *met1* that we examined tended to match with at least one potential siRNA-generating locus (Figure S4A). Thus, our observations suggest that some paralogous genes might be cryptic targets of RdDM, and become methylated only in *met1*. This does not seem to be due to an increase of small RNAs at these sites in *met1* (Figure S5D). One possibility is that a decrease in H3K27m3 marks could contribute to the onset of siRNA-directed DNA methylation (RdDM), after which methylation would then be maintained by H3K9m2-CMT3 feed-forward loop.

### Ectopic H3K9m2 and DNA methylation at PcG target genes is associated with a decrease of H3K27m3 marks, which specifically relocalize to *met1-*induced DNA hypomethylated regions

**Figure 4:**
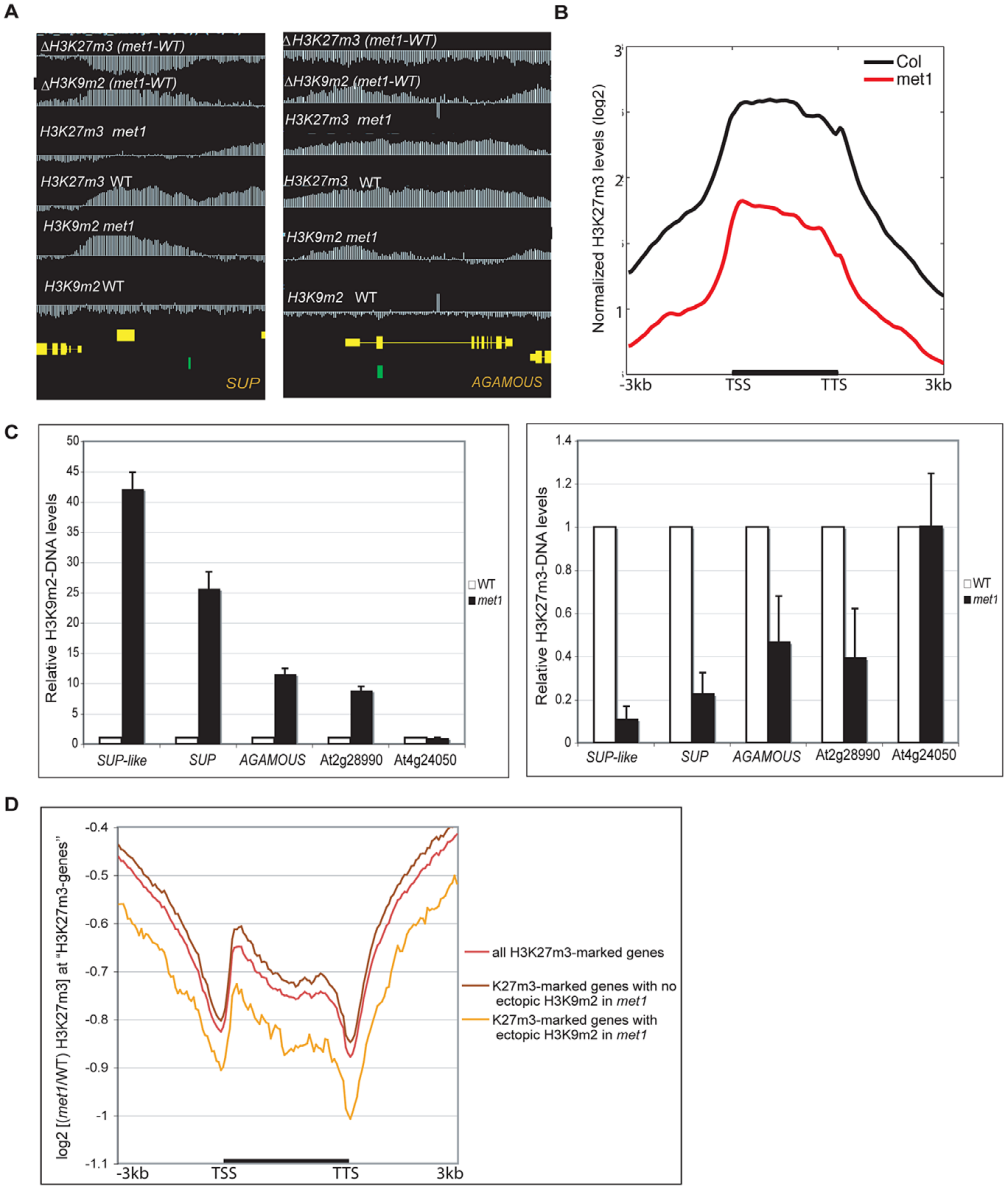
Gain of ectopic H3K9 dimethylation at PcG targets is associated with loss of H3K27m3 in *met1*. A. Representative views of SUPERMAN and AGAMOUS and H3K9m2 and H3K27m3 normalized signals at these loci. Both genes lose H3K27m3 in *met1*; this loss is stronger at the most H3K9m2 hypermethylated regions B. Average distribution of the normalized H3K27m3 signals in wild-type and *met1* across protein-coding genes that display H3K27m3 in wild type and gain H3K9m2 in *met1*. C. Analysis of H3K9m2 (left) and H3K27m3 (right) at various PcG targets by ChIP followed by real-time PCR. *SUP-like* is At2g42410 (ZFP11), which is highly homologous to *SUP*. Data were normalized to input DNA and to an internal control (actin). The average of two independent ChIP-experiments is shown. D. log2 *met1*/WT ratio of average H3K27m3 normalized values at H3K27m3-marked genes (i.e. PcG targets in wild type) referred here as “H3K27m3- genes”.

**Figure 5:**
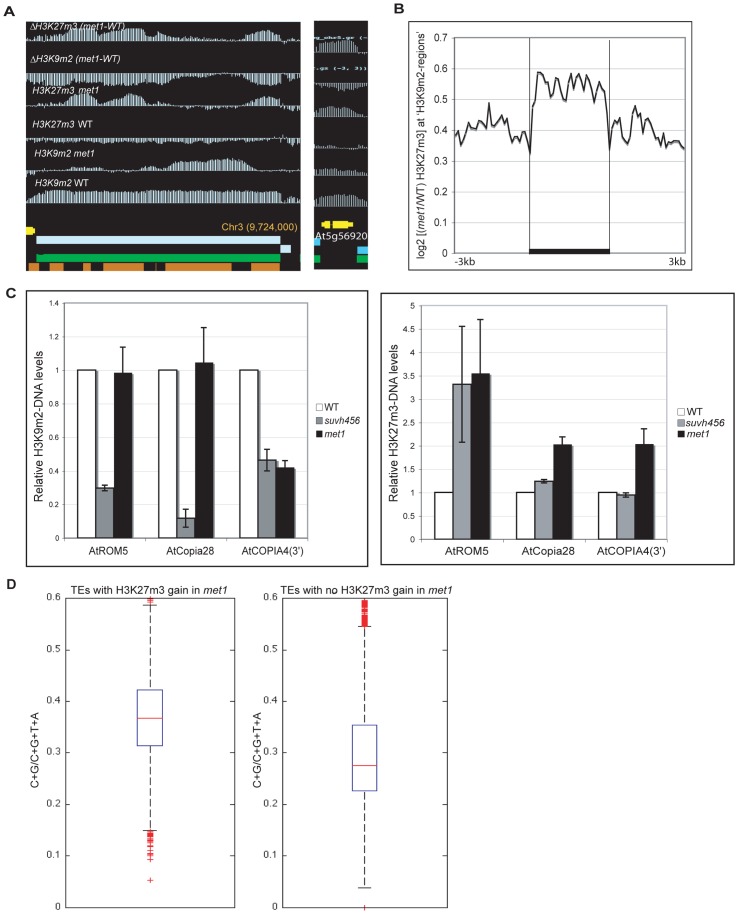
Ectopic gain of H3K27m3 at DNA hypomethylated regions. A. Genome-browser views of a transposable element on chromosome 3 (left) and of a heterochromatic gene, At5g56920 (right). B. Average distribution of the normalized H3K27m3 signals across H3K9m2 regions (horizontal black bars) in wild-type (defined in [9], perfectly correlate with transposons and methylated genes) C. Analysis of H3K9m2 and H3K27m3 marks at three transposons by ChIP followed by real-time PCR. Data were normalized to the input DNA and to an internal control (actin gene). D. C+G density of transposons that gain or do not gain H3K27m3 in *met1*. The red line represents the median; the edges of the box represents the 25th and 75th percentiles, the whiskers stretch out to 1.5× interquartile range above and below the edges of the box; the red dots represent the outliers. Two-tailed p-values were calculated by Wilcoxon ranksum test.

Since ectopic H3K9m2 and DNA hypermethylation in *met1* occurs at PcG targets, and PcG targets are usually non-overlapping with siRNAs and H3K9m2 [4], we sought to test whether H3K27m3 levels are reduced in *met1* at these loci. To this end, we generated high-resolution genome-wide maps of H3K27m3 in rosette-stage *met1* mutant plants and observed a massive redistribution of H3K27m3 levels throughout the genome. H3K27m3 levels were significantly decreased at ectopically H3K9m2 hypermethylated genes (Figure 4, Figure S9) and were significantly increased at H3K9m2 hypomethylated regions (Figure 5A and 5B, Figure S9), namely transposons and other repetitive DNA elements. The extent of the H3K27m3 decrease at H3K9m2 hypermethylated regions was correlated with the extent of H3K9m2 ectopic methylation at the genes tested by quantitative PCR (Figure 4C). However, we also observed that many PcG-targeted genes with limited or no ectopic H3K9m2 were also partially depleted of H3K27m3 marks in *met1*, but on average the decreases in H3K27m3 at these genes seemed smaller than observed for PcG-targeted genes that gained H3K9m2 and DNA methylation (Figure 4D, Figure S6A). A possibility is that the smaller decreases in H3K27m3 at these loci could result from the partial relocation of H3K27m3 marks and/or PcG complexes to transposable elements and heterochromatic genes (Figure 5A, Figure 5B, Figure S8). In addition, the massive increase of H3K27m3 marks at transposons is likely contributed by a global increase of H3K27m3 marks (Figure S6B) and of PcG gene expressions. According to our RNA-seq data, in *met1*, FIE, CLF and SWN expression levels are increased by 33% (P = 0.005), 13% (P = 0.33), and 32% (P<0.001), respectively).

An increase in H3K27m3 marks was previously observed at discrete heterochromatic loci and at chromocenters in *met1* [6] and our data show that this phenomenon can now be extended to hundreds of sites throughout the genome (Table S3). These findings suggest a model in which H3K9m2 and/or associated DNA methylation excludes H3K27m3 from heterochromatic loci in wild-type plants. To better understand the contributions of DNA and H3K9m2 methylation on H3K27m3 exclusion, we compared the pattern of H3K27m3 marks at several well-characterized transposable elements in various mutant backgrounds. Transposable elements such as *ROMANIAT5*, *AtCOPIA28* lost H3K9m2 marks in the triple *suvh4 suvh5 suvh6* (*suvh456*) mutant (in which both H3K9m2 and CHG methylation are reduced drastically, but not CG methylation) but H3K9m2 was not lost at these sites in the *met1* mutant (Figure 5C). However, we observed that the *met1* mutant exhibited a stronger increase in H3K27m3 marks at these sites compared to *suvh456* (Figure 5C). In addition, at AtCOPIA4 (in its 3′half), H3K9m2 levels were reduced to the same extent in both *suvh456* and *met1*, yet only *met1* gained H3K27m3 at this locus (Figure 5C). Together, these results were consistent with previous data in the *suvh4* mutant [6] and suggest that the loss of DNA methylation at heterochromatic loci in *met1*, rather than the loss of H3K9m2 marks, is associated with an increase in H3K27m3. This idea is further supported by the chromosomal distributions of H3K9m2 and H3K27m3 in *met1* which show that H3K27m3 is targeted to centromeric sites that are free of CG methylation in this background but still contain similar levels of H3K9m2 or even increased levels of H3K9m2 (for example transposons behaving like Class I genes) (Figure S8).

Notably, there was no consistent gain of H3K27m3 at *AtMU1* and at *AtCOPIA4* (in its 5′half) (Figure S7) suggesting that loss of CG methylation and associated H3K9m2 alone was not sufficient to induce H3K27m3 deposition. A recent study suggested that a high density of unmethylated CpG sites could be sufficient for vertebrate Polycomb recruitment [33]. Consistent with this idea, we found that the density of CG sites was higher at transposable elements that gained H3K27m3 in *met1* (Figure 5D) (including *AtCOPIA28*, *ROMANIAT5*, *AtCOPIA4*-3′half) than the ones that did not (including *AtMu1* and *AtCOPIA*-5′half). Thus, CG density may contribute to the differential recruitment of PcG complexes to transposons in *met1*.

### Transcriptional impact of the replacement of DNA/H3K9 methylation by H3K27m3 marks and vice-versa

**Figure 6:**
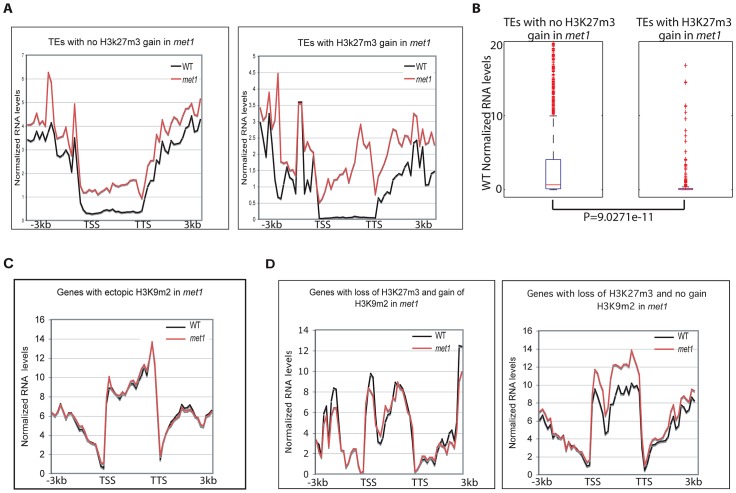
Genome-wide expression analyses. A. Transposable element expression levels in WT and *met1* backgrounds (represented as RPKM). B. Wild-type expression levels of transposable elements that gain or do not gain H3K27m3 in *met1*. The red line represents the median; the edges of the box represents the 25th and 75th percentiles, the whiskers stretch out to 1.5× interquartile range above and below the edges of the box; the red dots represent the outliers. Transposable elements with RPKM = 0 were removed when generating boxplots. Two-tailed p-values were calculated by Wilcoxon ranksum test. C. Expression levels of protein-coding genes in WT and *met1* at H3K9m2 hypermethylated genes. D. Expression levels of protein-coding genes in WT and *met1* at H3K27m3-marked genes.

To gain insight into the biological significance of the relocation of H3K27m3 to heterochromatic loci and of H3K9m2/DNA methylation to PcG genes, we performed RNA-seq in wild type and *met1* plants. Consistent with previous locus-specific analyses [6], transposable elements were usually reactivated in *met1*, despite the presence of ectopic H3K27m3 (Figure 6A). Therefore, H3K27m3 is not as competent as CG methylation and associated H3K9m2 in the silencing of transposons. Notably, the transposable elements targeted by H3K27m3 in *met1* tend to be lowly expressed in wild type (Figure 6A, Figure 6B). Further analyses of transposon expression in a *fie-met1* double mutant will be required to determine whether H3K27m3 marks can at least partially compensate for the loss of CG and H3K9m2 methylation in *met1* and whether the increase of H3K27m3 at these sites is a back-up mechanism deployed by the plant cell to avoid massive transposon expression and transposition.

We found that H3K9m2 hypermethylated loci generally did not alter expression levels (Figure 6C). This was also true at PcG-genes despite a significant loss of H3K27m3 marks (Figure 6D). This finding suggests a functional redundancy between H3K9m2 and H3K27m3 at PcG-targets in leaves, even though the dynamics of these two silencing marks are thought to be quite distinct, with H3K27m3 marks acting in a reversible manner during the course of development to allow developmental switches [5] and H3K9m2 acting in a permanent manner to lock a gene or transposon into a silent heterochromatic state. With these differences in epigenetic plasticity in mind, we propose that the replacement of H3K27m3 by H3K9m2 at PcG-targets may contribute to array of developmental defects, including many floral defects, which are observed in the *met1* mutant by locking PcG-target genes into a stably silent state, which is then unresponsive to developmental cues. Alternatively, the reactivation of key PcG-targets that lose H3K27m3 and become reactivated in *met1* could also contribute the developmental defects, reminiscent of those displayed in the *lhp1* mutant where H3K27m3-mediated silencing is impaired.

## Discussion

In *Arabidopsis*, loss of the maintenance CG DNA methyltransferase, MET1, results in DNA and H3K9 hypermethylation at some specific loci and in hypomethylation at other regions. Despite the strong parallels between this epigenetic state and those described in numerous cancers, how these patterns are established in *met1* and their biological significance has remained unknown. In this study, we analyzed the genome-wide patterns of ectopic H3K9m2 methylation (which mirrors DNA methylation) in the otherwise globally DNA hypomethylated *met1* mutant and provide significant insights into these questions. Based on our findings, we propose a model that accounts for the ectopic DNA and H3K9m2 methylation observed in *met1* mutant. First, genes that gain DNA and H3K9 methylation in *met1* fall into three categories: (1) a small class of genes that are affected by both *met1* and *ibm1* that are presumably IBM1 targets sensitive to the reduced levels of IBM1 expression in the *met1* background, (2) genes that possess low levels of H3K9m2 in wild type plants and become H3K9 hypermethylated in *met1* and (3) PcG-target genes (i.e. H3K27m3 pre-marked genes) that lose H3K27m3 marks in *met1*, but gain H3K9m2 marks. Interestingly, concomitant with the decrease in H3K27m3 at PcG targets, H3K27m3 levels increase at transposons and other heterochromatic loci where unmethylated CG sites may facilitate the recruitment of PcG complexes. The replacement of CG DNA methylation by H3K27m3 in *met1* and vice-versa suggests that these two marks are mutually exclusive in *Arabidopsis*, as previously demonstrated in mammals at some imprinted loci [34] as well a in cancer cells [35]. While the exclusion of H3K27m3 by DNA methylation was previously proposed in *Arabidopsis* [6], [7], our findings add strength to this assertion and further suggest that it is the loss of H3K27m3 at PcG targets that contributes to the occurrence of H3K9m2 and DNA hypermethylation. This causative relationship is supported by the observation that the H3K27m3 decrease is not specific to H3K9m2 and DNA hypermethylated genes although it is stronger at these loci, consistent with the notion of mutual exclusion.

Interestingly, the PcG-target genes that become H3K9m2 and DNA hypermethylated may also represent cryptic targets of RdDM as they tend to have higher than average levels of sequence homology with other regions in the genome, many of which are known to generate siRNAs. Many genes such as transcription factors are part of large families and the presence of H3K27m3 at these loci may have the dual role of mediating transient, reversible repression and excluding RdDM and associated H3K9m2 methylation. How Polycomb-complexes are recruited to deposit H3K27m3 marks is still unknown in *Arabidopsis* [5]. However, at the FLC locus, there are *cis*-sequences that have been shown to be important for the recruitment of PRC2 (Polycomb Repressive Complex 2) through a long-coding RNA [36], [37]. Our data suggest that a high density of unmethylated CG sites, as previously observed in vertebrates, may be another factor facilitating PcG recruitment (Figure 5D). Further analyses may identify *cis* sequences in the transposons targeted by PRC2 in *met1* and/or show a general role for non-coding RNAs in PRC2 recruitment. Alternatively, heavily methylated CG sites such as those seen in transposons, could recruit a H3K27m3 demethylase which would be inactive in *met1*. Future mechanistic exploration of these new epigenetic phenomena in *met1* will likely bring insight into the recruitment of PcG complexes as well as RdDM components.

Our observations also provide a possible explanation for the drastic developmental phenotypes displayed by the *met1* mutant: genes targeted by ectopic DNA and H3K9m2 methylation in *met1* are PcG-targets in wild type plants, which are enriched in genes involved in transcriptional regulation and development. At specific developmental stages, for example during the vegetative phase where our analyses were performed, it appears that H3K9m2 marks are functionally redundant with H3K27m3 marks since the vast majority of genes with either mark in this study remained silent. However, the replacement of a transient repressive mark such as H3K27m3 by a stable silencing mark such as H3K9m2 may affect gene transcription during specific developmental windows where H3K27m3 marks are removed, thus impairing critical developmental switches and contributing to the myriad of developmental phenotypes observed in *met1* mutants. Furthermore, the finding that hypomethylated regions of the genome induced by loss of MET1 in vegetative tissue can become targets of the Polycomb silencing machinery raises the question of whether hypomethylation of the genome caused by other processes also leads to PcG-targeting and gene silencing. Several examples of naturally occurring global hypomethylation have recently been described. These include the endosperm (plant extra-embryonic tissues), which is globally hypomethylated, due to the activity of the DNA demethylase DEMETER but also possibly due to down-regulation of MET1 in this context [38]. Interestingly, H3K27m3 was found at DNA hypomethylated transposable elements and genes that had less CG methylation than in the vegetative tissues [7]. In this respect, the strong endosperm phenotype observed after loss of polycomb function (proliferation and eventually seed abortion [39] [40]) could indicate the crucial role of H3K27m3 marks at DNA hypomethylated sites. In addition, other studies revealed DNA hypermethylation (presumably associated with H3K9m2 hypermethylation) of specific sites in the endosperm [41]. However, the nature of these sites has not been investigated and it is possible that PcG-targets in the endosperm are similarly affected as in the vegetative tissues of *met1* mutants.

Finally, our work in an *Arabidopsis* globally DNA hypomethylated mutant has uncovered striking similarities with epigenetic phenomena occurring in human cancer cells. First, H3K9m2 and DNA hypermethylated promoters in human cancer cells tend to be marked with H3K27m3 in the corresponding adult cells or in the progenitor cells they are derived from. Another point of convergence is the decrease of H3K27m3 marks associated with the ectopic gain of H3K9m2 in both contexts [42]. Finally, repressive chromatin formation, mediated in particular by H3K27m3, was observed at DNA hypomethylated regions in breast cancer cells [35]. The same study demonstrated genome-wide, mutual exclusivity of these two marks, which had been previously shown at one imprinted locus in mouse [34]. The striking similarities between the epigenetic landscapes of a globally hypomethylated mutant, the globally hypomethylated endosperm and human cancer cells suggest common underlying mechanisms, and suggests the potential of future *Arabidopsis* research as a framework for understanding developmental and cancer biology.

## Materials and Methods

We used the *met1-3* allele [43] and the SALK_006042 line for isolating *ibm1* mutants. *met1*-1^st^ generation homozygous mutants and *ibm1*-1^st^ generation homozygous mutants were isolated from a segregating population by genotyping. *met1*-2^nd^ generation second mutants are the progeny of a single *met1-*1^st^ generation homozygous mutant that was partially fertile. The entire shoots of 3 weeks old Arabidopsis plants (Col-0 ecotype), grown for 3 weeks under continuous light, were harvested, cross-linked as described in [9], frozen under liquid nitrogen and grown to powder (2 g). *Arabidopsis* chromatin enriched for H3K9m2 and H3K27m3 was immunoprecipitated using an antibody that specifically recognizes H3K9m2 (Abcam 1220) and an antibody that specifically recognizes H3K27m3 (Upstate 07-449) respectively. Unmodified H3 was also immunoprecipitated (Abcam 1791-100). H3 ChIP and input DNA were used as controls. ChIP, DNA purification and amplification were performed as in [9]; Roche Nimblegen performed labelling and hybridization of the samples, washing and scanning.

All ChIP signals were normalized with either H3 ChIP or input genomic DNA by taking the log2 ratio and adjusted so that the average log2 ratio score across the genome was zero. For each mark, four independent ChIP experiments on four different biological sample replicates were performed. The two first independent ChIPs were pooled and used to generate libraries and for subsequent chip-hybridization. The two other independent ChIPs were used for validation of the ChIP-chip data by qPCR. qPCR was performed in duplicates or triplicates. H3K9m2 hypermethylated regions were defined by using BLOC [26]. The log2 ratio of mutant to wild-type scores were taken, Z-score transformed, and a cutoff of 0.75 was applied for all *met1* datasets, and 0.8 was applied for all *ibm1* datasets. The cutoffs were determined based on visually examining genome-wide data and validation experiments, to minimize false-positives but also to avoid missing truly hypermethylated genes. The choice of two different cutoffs for the *ibm1* and *met1* analyses presumably results from a slight difference in the ChIP efficiencies between the two experiments. All sets of defined regions were significant (FDR<0.01). To define H3K27m3 enriched genes, H3K27m3 enriched regions were identified. The genome was tiled into 200 bp bins (100 bp overlap) and z-scores were calculated. A Z>2 cutoff was applied, and regions within 200 bp were merged. Genes that overlapped by at least 150 bp were defined to be H3K27m3 enriched genes. Genes that became H3K27m3 hypermethylated in *met1* were defined by calculating the log2 ratio of mutant to wild-type scores in 200 bp bins (100 bp overlap), Z-score transformed, and Z>2 cutoff was applied. Regions within 200 bp were merged, and finally only regions >500 bp in size were selected. Genes that overlapped by at least 150 bp were defined to be H3K27m3 hypermethylated genes in *met1*. Genes that became H3K27m3 hypomethylated in *met1* were defined in a similar matter as hypermethylated genes, except that a Z<-3 cutoff was applied.

Plants grown in the same conditions were used for RNA extraction using a standard protocol. The RNA of two independent biological sample replicates was extracted and pooled for RNA-seq analysis, and a third replicate used to validate the data by RT-qPCR (performed in duplicates or triplicates). RNA-seq libraries were generated following the manufacturer instructions (Illumina). DNA methylation analyses were performed using published whole genome bisulfite sequencing data and RNA-seq data [16]. Tandem-repeats were defined in [17], dispersed repeats were defined in [16] and H3K9m2-regions were defined in [9].

Plants grown in the same conditions were used for Histone Western Blot experiments. Chromatin was extracted as was performed for ChIP. 30 ug and 10 ug of proteins were loaded to detect H3K27m3 and histone H3 respectively, and the antibodies used for ChIP experiments were used for detection.

### Data access

http://www.ncbi.nlm.nih.gov/geo/query/acc.cgi?token=dvotficqwuacufg&acc=gse37075

## Supporting Information

Figure S1 Genes showing ectopic H3K9m2 in *met1* in this study are DNA hypermethylated in *Lister et al.* study. Representative views of genes that gain H3K9m2 marks in their coding-region in *met1* mutants. Representative views (left) and screenshots of the AnnoJ Arabidopsis epigenome browser (left) are shown. (http://neomorph.salk.edu/epigenome/epigenome.html). Yellow horizontal bars: protein-coding genes; blue horizontal bars: transposable elements, green bars: dispersed repeats (i.e. regions of sequence homology); vertical blue bar: relative H3K9m2 levels.
(PDF)

Figure S2 Representative views of genes that gain H3K9m2 marks in their body in both *ibm1* and *met1* mutants. We observed that H3K9m2 hypermethylation in *met1* was usually not as extensive as in *ibm1* in accordance with the idea that these genes may be targets of IBM1, the levels of which are reduced in *met1*. Yellow horizontal bars: protein-coding genes; blue horizontal bars: transposable elements; vertical blue bar: relative H3K9m2 levels.
(PDF)

Figure S3 Supplementary information on Class I genes. A. Verification of H3K9m2 states at genes pre-marked with H3K9m2 in wild type that gain H3K9m2 in *met1* (Class I genes) by independent chromatin immunoprecipitation experiments. The immunoprecipitated DNA corresponding to a Class I gene (At3g54590) and a transposable element (*TA3*), shown to be hypermethylated in the ChIP-chip analysis, was quantified by real-time PCR and normalized to the input DNA and to an internal control (actin gene). Class I genes that gain H3K9m2 in *met1* have the same response to *met1* mutation as some transposable elements such as *TA3*, where H3K9m2 is highly dependant on non-CG methylation. Genome-browser views of these loci are shown on the right. Yellow horizontal bars: protein-coding genes; blue horizontal bars: transposable elements; green bars: dispersed repeats (regions with sequence of homology); orange bars: small RNAs clusters (MPSS); purple bars: tandem repeats B. Fraction of H3K9m2 hypermethylated genes in *met1* that contain a transposable element. C. Representative views of small RNA accumulation at Class I genes in wild type and *met1* (AnnoJ, http://neomorph.salk.edu/epigenome/epigenome.html). The ‘translucid’ reads indicate mapping to multiple locations.
(PDF)

Figure S4 Supplementary information on Class II genes. A. Representative genome-browser views of genes (yellow bars) that gain H3K9m2 marks in their coding-region in *met1* and are pre-marked with abundant H3K27m3 in WT. The green bars represent ‘dispersed repeats’ which show that these genes (in each box) present sequence homology to each other. Left panels: genes homologous to At2g28990 (by their Leucine-rich kinase domains) either linked (AT2g29000, Blast Score to At2g28990 of 5e-29) or unlinked (At1g51805 and At3g21340, Blast Score to At2g28990 of 4e-48 and 3e-67, respectively). AT1g51805 is potential siRNA-generator locus as shown by the presence of tandem repeats (purple bars) and small RNAs (orange bars). Top right panel: At4g08990 is a potential siRNA generator locus for At5g49160. Bottom left panel: SCPL genes; At1g43780 generates siRNAs. Middle right panel: in this example, all the loci shown (glycosyl-hydrolase genes) can produce siRNAs and could potentially target each other in trans in *met1*. B. Verification of H3K9m2 states at Class II genes by independent chromatin immunoprecipitation experiments. The immunoprecipitated DNA was quantified by real-time PCR and normalized to the input DNA and to an internal control (actin gene). For paralogous genes, only primers that hybridize to gene-specific sequences were used.
(PDF)

Figure S5 Supplementary information on Class II genes. A. Representative views of genes that gain H3K9m2 in *met1* and are H3K27m3-marked in wild type; however, these genes were not recovered among Class II genes due to their low levels of H3K27m3 in wild-type. This shows that the number of true Class II genes (H3K27m3 in wild type and ectopic H3K9m2 in *met1*) maybe underestimated by our stringencies. B. Representative views of genes that are marked with H3K27m3 in WT but do not gain ectopic H3K9m2 in *met1*, suggesting that there are additional features that contribute to ectopic H3K9m2 in *met1*. C. Representative genome-browser views of two paralogous or related genes that were not retrieved as dispersed repeats by ‘Repeat Masker’, CITRATE SYNTHASE 1 and 2 genes (At3g58740 and At3g58750). These examples suggest that in the genome-wide analysis shown in Figure 3E, the proportion of genes H3K9m2 hypermethylated in *met1* that correspond to duplicated/paralogous/related genes is underestimated and must be larger than 40%. Yellow horizontal bars in genome browser views: protein-coding genes; blue horizontal bars: transposable elements; green bars: dispersed repeats (regions with sequence of homology); orange bars: small RNAs clusters (MPSS). D. Average distribution of small RNA-seq reads [16] across Class II genes.
(PDF)

Figure S6 Supplementary information on H3K27m3 changes in *met1*. A. Representative views showing PcG-target genes that do not gain H3K9m2 in *met1*, yet lose H3K27m3 marks. B. Global accumulation of H3K27m3 marks in WT and *met1* mutants by Western Blot (upper panel). Detection of histone H3, independently of its modifications, is shown as a loading control (lower panel).
(PDF)

Figure S7 Analysis of H3K9m2 and H3K27m3 marks at two transposons by ChIP followed by real-time PCR. Data were normalized to the input DNA and to an internal control (actin gene).
(PDF)

Figure S8 Chromosomal distributions of H3K27m3 (left) and H3K9m2 (right) regions. Left and right panels show the normalized levels of H3K27m3 and H3K9m2 respectively in a sliding 100 kilobase windows. The black arrow indicates the centromeric regions. The blue arrow indicates the heterochromatic knob on chromosome 4.
(PDF)

Figure S9 Supplementary information on ChIP-chip validation by ChIP-qPCR. Data shown in Figure 4C and Figure 5C are normalized to input, with actin shown as a separate control so that ChIP efficiencies can be visualized.
(PDF)

Table S1 List of genes that gain H3K9m2 marks in *met1* mutants. Genes that already have lower levels of H3K9m2 in WT (‘class I genes’) are separated from genes that do not have H3K9m2 marks in WT.
(XLSX)

Table S2 List of genes that gain H3K9m2 marks in *ibm1*.
(XLSX)

Table S3 List of transposable elements and genes that gain H3K27m3 marks in *met1*.
(XLSX)
