# Supplementary material for: Loss of the DNA Methyltransferase MET1 Induces H3K9 Hypermethylation at PcG Target Genes and Redistribution of H3K27 Trimethylation to Transposons in Arabidopsis thaliana
Source: PLoS Genet. 2012 Nov 29;8(11):e1003062. doi: 10.1371/journal.pgen.1003062 (PMC3510029; doi:10.1371/journal.pgen.1003062)
Supplement: Figure S1 — Genes showing ectopic H3K9m2 in met1 in this study are DNA hypermethylated in Lister et al. study. Representative views of genes that gain H3K9m2 marks in their coding-region in met1 mutants. Representative views (left) and screenshots of the AnnoJ Arabidopsis epigenome browser (left) are shown. (http://neomorph.salk.edu/epigenome/epigenome.html). Yellow horizontal bars: protein-coding genes; blue horizontal bars: transposable elements, green bars: dispersed repeats (i.e. regions of sequence homology); vertical blue bar: relative H3K9m2 levels. (PDF) [file pgen.1003062.s001.pdf]

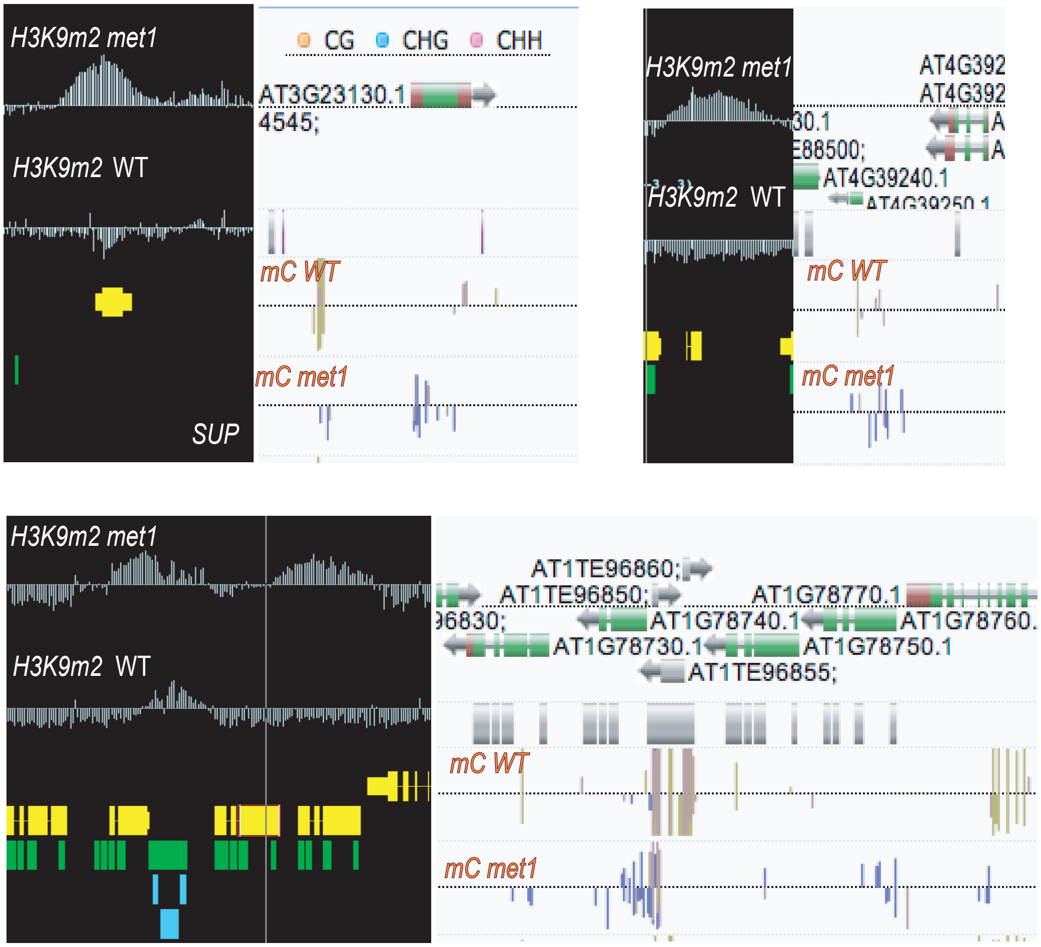

Supplemental Figure 1. Genes showing ectopic H3K9 dimethylation in this study are DNA hypermethylated in *Lister et al.* study. Representative views of genes that gain H3K9m2 marks in their coding-region in *met1* mutants. Representative IGB views (left) and screenshots of the AnnoJ Arabidopsis epigenome browser (http://neomorph.salk.edu/epigenome/epigenome.html) (left) are shown. Yellow horizontal bars: protein-coding genes; blue horizontal bars: transposable elements, green bars: dispersed repeats (i.e. regions of sequence homology); vertical blue bar: relative H3K9m2 levels.
