# Supplementary material for: Loss of the DNA Methyltransferase MET1 Induces H3K9 Hypermethylation at PcG Target Genes and Redistribution of H3K27 Trimethylation to Transposons in Arabidopsis thaliana
Source: PLoS Genet. 2012 Nov 29;8(11):e1003062. doi: 10.1371/journal.pgen.1003062 (PMC3510029; doi:10.1371/journal.pgen.1003062)
Supplement: Figure S2 — Representative views of genes that gain H3K9m2 marks in their body in both ibm1 and met1 mutants. We observed that H3K9m2 hypermethylation in met1 was usually not as extensive as in ibm1 in accordance with the idea that these genes may be targets of IBM1, the levels of which are reduced in met1. Yellow horizontal bars: protein-coding genes; blue horizontal bars: transposable elements; vertical blue bar: relative H3K9m2 levels. (PDF) [file pgen.1003062.s002.pdf]

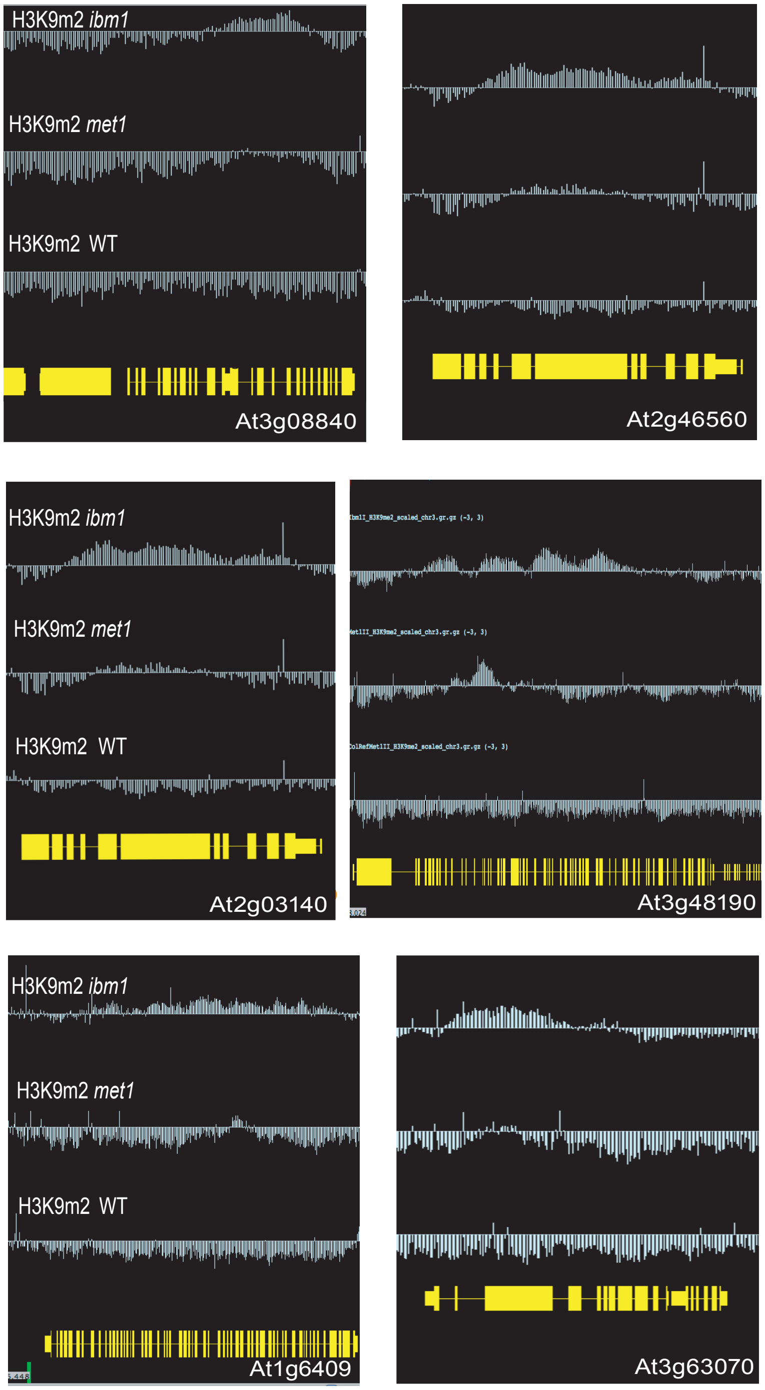

Supplemental Figure 2. Representative views of genes that gain H3K9m2 marks in their body in both *ibm1* and *met1* mutants. We observed that H3K9m2 hypermethylation in *met1* was usually not as extensive as in *ibm1* in accordance with the idea that these genes may be targets of IBM1, the levels of which are reduced in *met1*.Yellow horizontal bars: protein-coding genes; blue horizontal bars: transposable elements; vertical blue bar: relative H3K9m2 levels.
