# Supplementary material for: Loss of the DNA Methyltransferase MET1 Induces H3K9 Hypermethylation at PcG Target Genes and Redistribution of H3K27 Trimethylation to Transposons in Arabidopsis thaliana
Source: PLoS Genet. 2012 Nov 29;8(11):e1003062. doi: 10.1371/journal.pgen.1003062 (PMC3510029; doi:10.1371/journal.pgen.1003062)
Supplement: Figure S3 — Supplementary information on Class I genes. A. Verification of H3K9m2 states at genes pre-marked with H3K9m2 in wild type that gain H3K9m2 in met1 (Class I genes) by independent chromatin immunoprecipitation experiments. The immunoprecipitated DNA corresponding to a Class I gene (At3g54590) and a transposable element (TA3), shown to be hypermethylated in the ChIP-chip analysis, was quantified by real-time PCR and normalized to the input DNA and to an internal control (actin gene). Class I genes that gain H3K9m2 in met1 have the same response to met1 mutation as some transposable elements such as TA3, where H3K9m2 is highly dependant on non-CG methylation. Genome-browser views of these loci are shown on the right. Yellow horizontal bars: protein-coding genes; blue horizontal bars: transposable elements; green bars: dispersed repeats (regions with sequence of homology); orange bars: small RNAs clusters (MPSS); purple bars: tandem repeats B. Fraction of H3K9m2 hypermethylated genes in met1 that contain a transposable element. C. Representative views of small RNA accumulation at Class I genes in wild type and met1 (AnnoJ, http://neomorph.salk.edu/epigenome/epigenome.html). The ‘translucid’ reads indicate mapping to multiple locations. (PDF) [file pgen.1003062.s003.pdf]

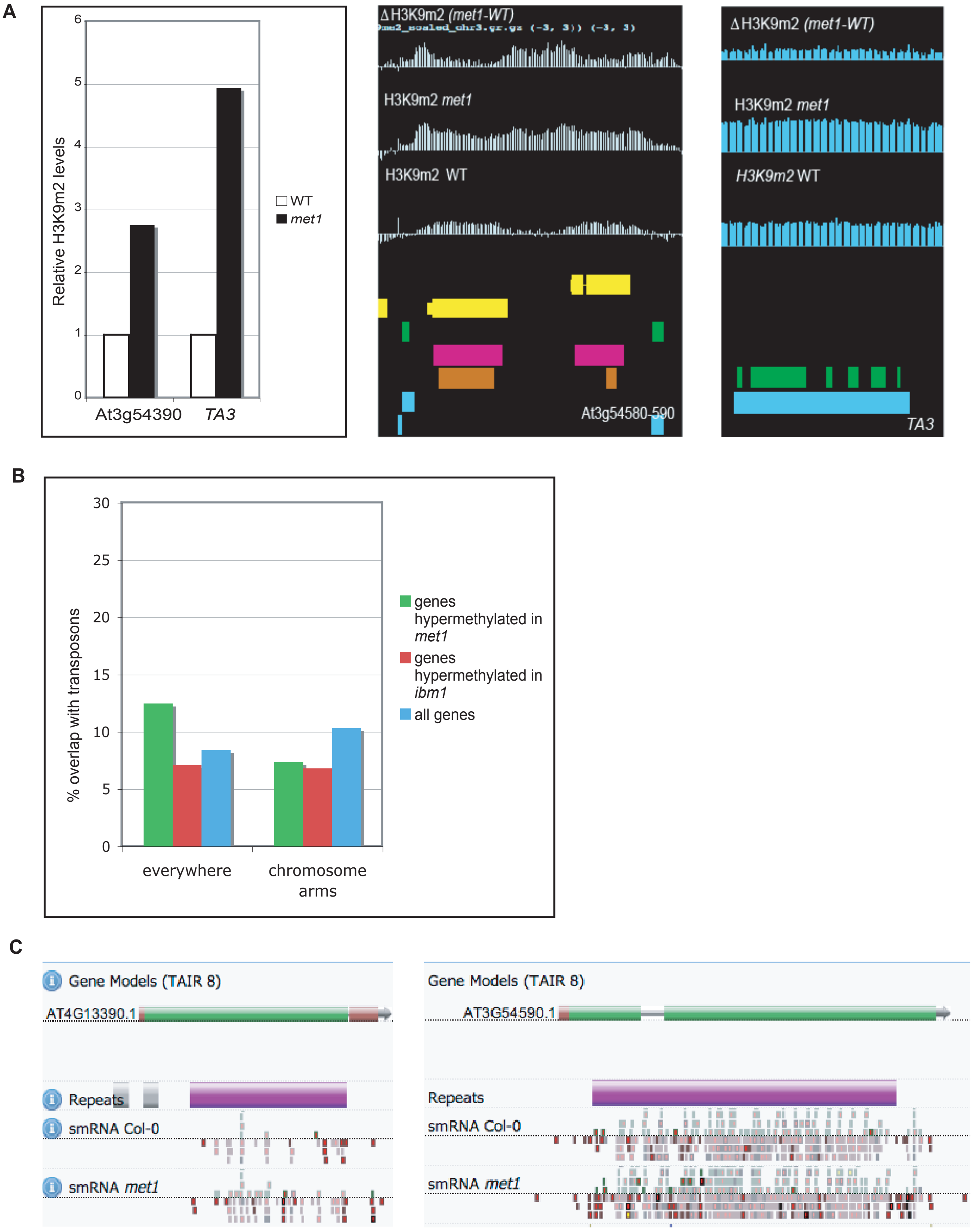

Supplemental Figure 3. Supplementary information on Class I genes. **A.** Verification of H3K9m2 states at genes pre-marked with H3K9m2 in wild type that gain H3K9m2 in *met1* (class I genes) by independent chromatin immunoprecipitation experiments. The immunoprecipitated DNA corresponding to a Class I gene (At3g54590) and a transposable element (*TA3*), -shown to be hypermethylated in the ChIP-chip analysis- was quantified by real-time PCR and normalized to the input DNA and to an internal control (actin gene). Class I genes that gain H3K9m2 in *met1* have the same response to *met1* mutation as some transposable elements such as *TA3*, where H3K9m2 is highly dependant on non-CG methylation. Genome-browser views of these loci are shown on the right. Yellow horizontal bars: protein-coding genes; blue horizontal bars: transposable elements; green bars: dispersed repeats (regions with sequence of homology); orange bars: small RNAs clusters (MPSS); purple bars: tandem repeats **B.** Fraction of H3K9m2 hypermethylated genes in *met1* that contain a transposable element (TE). **C.** Representative views of small RNA accumulation at Class I genes in wild type and *met1* (Arabidopsis Epigenome Browser, AnnoJ, http://neomorph.salk.edu/epigenome/epigenome.html). The 'translucid' reads indicate mapping to multiple locations.
