# Supplementary material for: Loss of the DNA Methyltransferase MET1 Induces H3K9 Hypermethylation at PcG Target Genes and Redistribution of H3K27 Trimethylation to Transposons in Arabidopsis thaliana
Source: PLoS Genet. 2012 Nov 29;8(11):e1003062. doi: 10.1371/journal.pgen.1003062 (PMC3510029; doi:10.1371/journal.pgen.1003062)
Supplement: Figure S4 — Supplementary information on Class II genes. A. Representative genome-browser views of genes (yellow bars) that gain H3K9m2 marks in their coding-region in met1 and are pre-marked with abundant H3K27m3 in WT. The green bars represent ‘dispersed repeats’ which show that these genes (in each box) present sequence homology to each other. Left panels: genes homologous to At2g28990 (by their Leucine-rich kinase domains) either linked (AT2g29000, Blast Score to At2g28990 of 5e-29) or unlinked (At1g51805 and At3g21340, Blast Score to At2g28990 of 4e-48 and 3e-67, respectively). AT1g51805 is potential siRNA-generator locus as shown by the presence of tandem repeats (purple bars) and small RNAs (orange bars). Top right panel: At4g08990 is a potential siRNA generator locus for At5g49160. Bottom left panel: SCPL genes; At1g43780 generates siRNAs. Middle right panel: in this example, all the loci shown (glycosyl-hydrolase genes) can produce siRNAs and could potentially target each other in trans in met1. B. Verification of H3K9m2 states at Class II genes by independent chromatin immunoprecipitation experiments. The immunoprecipitated DNA was quantified by real-time PCR and normalized to the input DNA and to an internal control (actin gene). For paralogous genes, only primers that hybridize to gene-specific sequences were used. (PDF) [file pgen.1003062.s004.pdf]

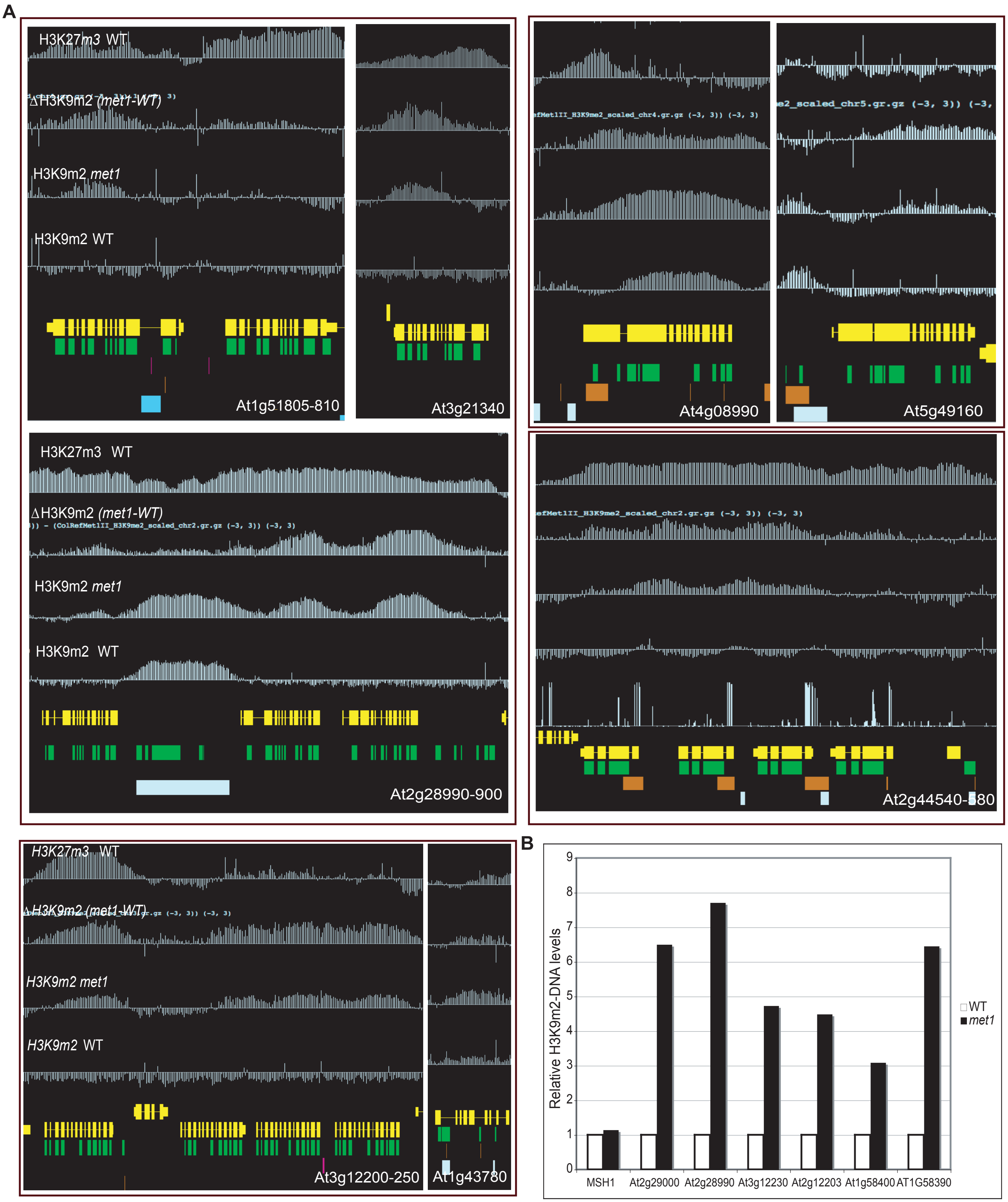

Supplemental Figure 4. Supplementary information on Class II genes. A. Representative genome-browser views of genes (yellow bars) that gain H3K9m2 marks in their coding-region in *met1* and are pre-marked with abundant H3K27m3 in WT. The green bars represent 'dispersed repeats' which show that these genes (in each box) present sequence homology to each other. Left panels: genes homologous to At2g28990 (by their Leucine-rich kinase domains) either linked (AT2g29000, Blast Score to At2g28990 of 5e-29) or unlinked (At1g51805 and At3g21340, Blast Score to At2g28990 of 4e-48 and 3e-67 respectively). AT1g51805 is potential siRNA-generator locus as shown by the presence of tandem repeats (purple bars) and small RNAs (orange bars). Top right panel: At4g08990 is a potential siRNA generator locus for At5g49160. Bottom left panel: SCPL genes; At1g43780 generates siRNAs. Middle right panel: in this example, all the loci shown (glycosyl-hydrolase genes) can produce siRNAs and could potentially target each other in trans in *met1*. **B.** Verification of H3K9m2 state at Class II genes by independent chromatin immunoprecipitation experiments. The immunoprecipitated DNA was quantified by real-time PCR and normalized to the input DNA and to an internal control (Actin gene). For paralogous genes, only primers that hybridize to gene-specific sequences were used.
