# Supplementary material for: Loss of the DNA Methyltransferase MET1 Induces H3K9 Hypermethylation at PcG Target Genes and Redistribution of H3K27 Trimethylation to Transposons in Arabidopsis thaliana
Source: PLoS Genet. 2012 Nov 29;8(11):e1003062. doi: 10.1371/journal.pgen.1003062 (PMC3510029; doi:10.1371/journal.pgen.1003062)
Supplement: Figure S5 — Supplementary information on Class II genes. A. Representative views of genes that gain H3K9m2 in met1 and are H3K27m3-marked in wild type; however, these genes were not recovered among Class II genes due to their low levels of H3K27m3 in wild-type. This shows that the number of true Class II genes (H3K27m3 in wild type and ectopic H3K9m2 in met1) maybe underestimated by our stringencies. B. Representative views of genes that are marked with H3K27m3 in WT but do not gain ectopic H3K9m2 in met1, suggesting that there are additional features that contribute to ectopic H3K9m2 in met1. C. Representative genome-browser views of two paralogous or related genes that were not retrieved as dispersed repeats by ‘Repeat Masker’, CITRATE SYNTHASE 1 and 2 genes (At3g58740 and At3g58750). These examples suggest that in the genome-wide analysis shown in Figure 3E, the proportion of genes H3K9m2 hypermethylated in met1 that correspond to duplicated/paralogous/related genes is underestimated and must be larger than 40%. Yellow horizontal bars in genome browser views: protein-coding genes; blue horizontal bars: transposable elements; green bars: dispersed repeats (regions with sequence of homology); orange bars: small RNAs clusters (MPSS). D. Average distribution of small RNA-seq reads [16] across Class II genes. (PDF) [file pgen.1003062.s005.pdf]

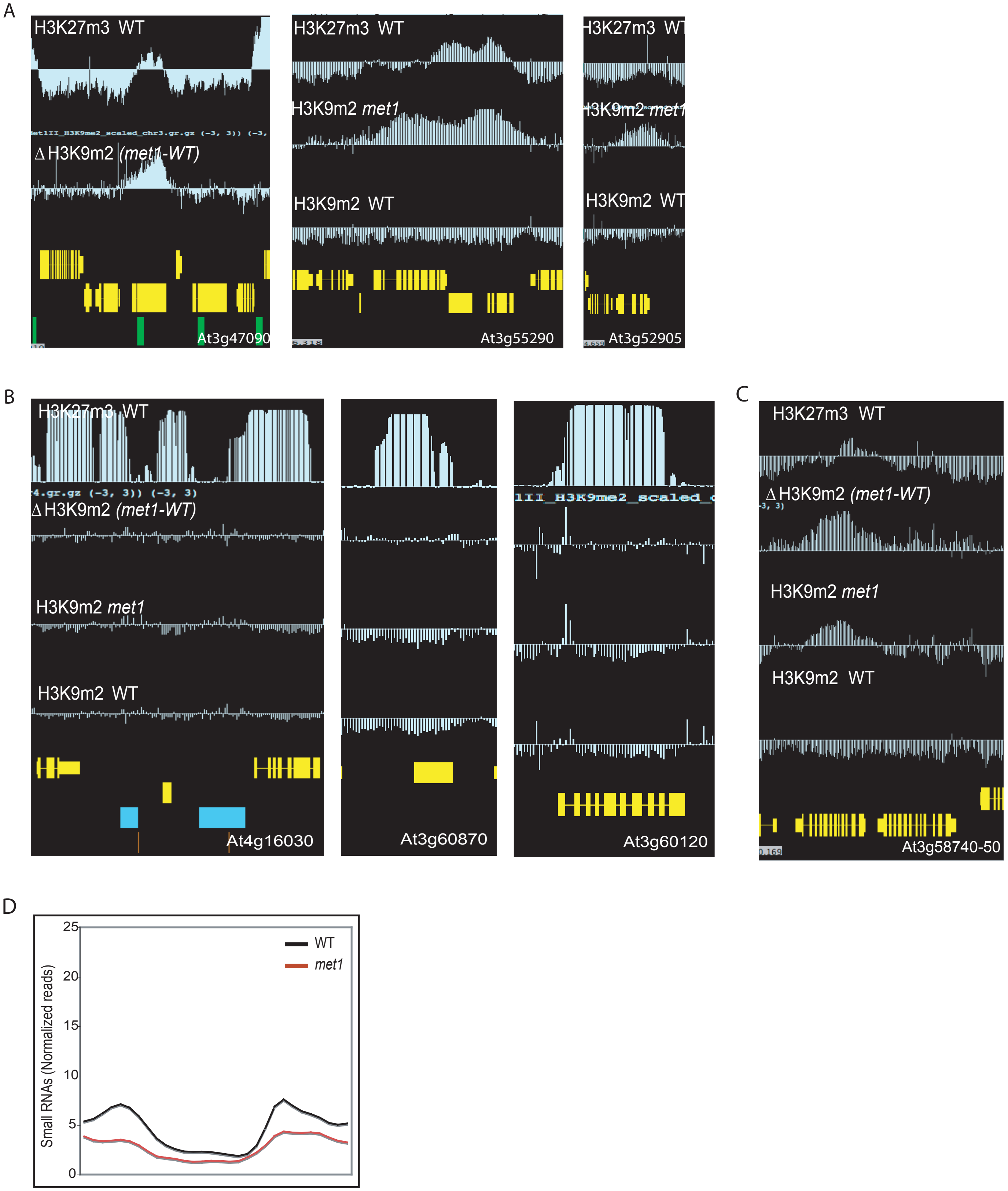

Supplemental Figure 5. Supplementary information on Class II genes. A. Representative views of genes that gain H3K9m2 in *met1* and are H3K27m3-marked in wild-type; however, these genes were not recovered among Class II genes due to their low levels of H3K27m3 in wild-type. This shows that the number of true Class II genes (H3K27m3 in wild type and ectopic H3K9m2 in *met1*) maybe underestimated by our stringencies. Representative IGB view of genes marked with H3K27m3 marks that do not gain ectopic H3K9m2 in *met1*. **B.** Representative views of genes that are marked with H3K27m3 in WT but do not gain ectopic H3K9m2 in *met1*, suggesting that there are additional features that contribute to ectopic H3K9m2 in *met1*. **C.** Representative genome-browser views of two paralogous or related genes that were not retrieved as dispersed repeats by 'Repeat Masker', CITRATE SYNTHASE 1 and 2 genes (At3g58740 and At3g58750). These examples suggest that in the genome-wide analysis shown in Figure 2F, the proportion of genes H3K9m2 hypermethylated in *met1* that correspond to duplicated/paralogous/related genes is underestimated and must be larger than 40%. Yellow horizontal bars in IGB views: protein-coding genes; blue horizontal bars: transposable elements; green bars: dispersed repeats (regions with sequence of homology); orange bars: small RNA clusters (MPSS). **D.** Average distribution of small RNA-seq reads [16] across Class II genes.

Supplemental Figure 5
