# Supplementary material for: Loss of the DNA Methyltransferase MET1 Induces H3K9 Hypermethylation at PcG Target Genes and Redistribution of H3K27 Trimethylation to Transposons in Arabidopsis thaliana
Source: PLoS Genet. 2012 Nov 29;8(11):e1003062. doi: 10.1371/journal.pgen.1003062 (PMC3510029; doi:10.1371/journal.pgen.1003062)
Supplement: Figure S6 — Supplementary information on H3K27m3 changes in met1. A. Representative views showing PcG-target genes that do not gain H3K9m2 in met1, yet lose H3K27m3 marks. B. Global accumulation of H3K27m3 marks in WT and met1 mutants by Western Blot (upper panel). Detection of histone H3, independently of its modifications, is shown as a loading control (lower panel). (PDF) [file pgen.1003062.s006.pdf]

A

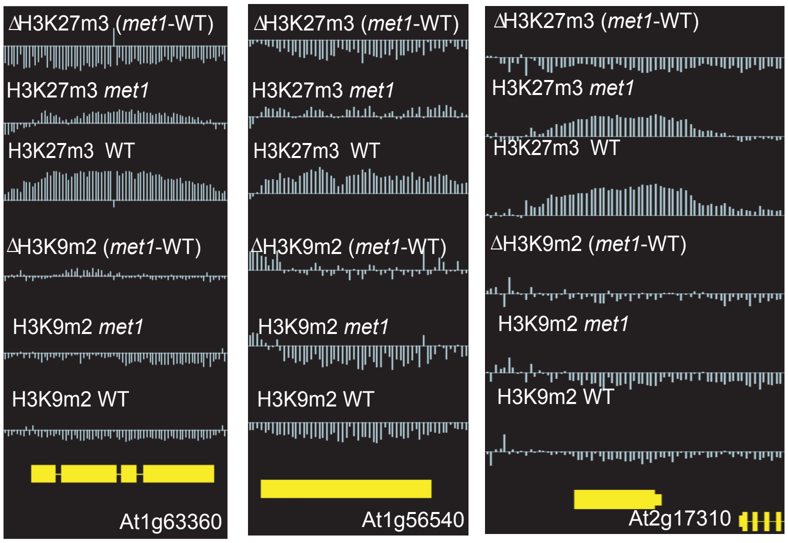

B

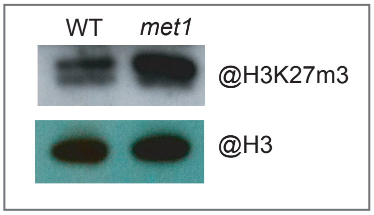

Supplemental Figure 6. Supplementary information on H3K27m3 changes in *met1*. A. Representative views showing PcG-target genes that do not gain H3K9m2 in *met1*, yet lose H3K27m3 marks. **B.** Global accumulation of H3K27m3 marks in WT and *met1* mutants by Western Blot (upper panel). Detection of histone H3 -independently of its modifications- is shown as a loading control (lower panel).
