# Supplementary material for: Loss of the DNA Methyltransferase MET1 Induces H3K9 Hypermethylation at PcG Target Genes and Redistribution of H3K27 Trimethylation to Transposons in Arabidopsis thaliana
Source: PLoS Genet. 2012 Nov 29;8(11):e1003062. doi: 10.1371/journal.pgen.1003062 (PMC3510029; doi:10.1371/journal.pgen.1003062)
Supplement: Figure S7 — Analysis of H3K9m2 and H3K27m3 marks at two transposons by ChIP followed by real-time PCR. Data were normalized to the input DNA and to an internal control (actin gene). (PDF) [file pgen.1003062.s007.pdf]

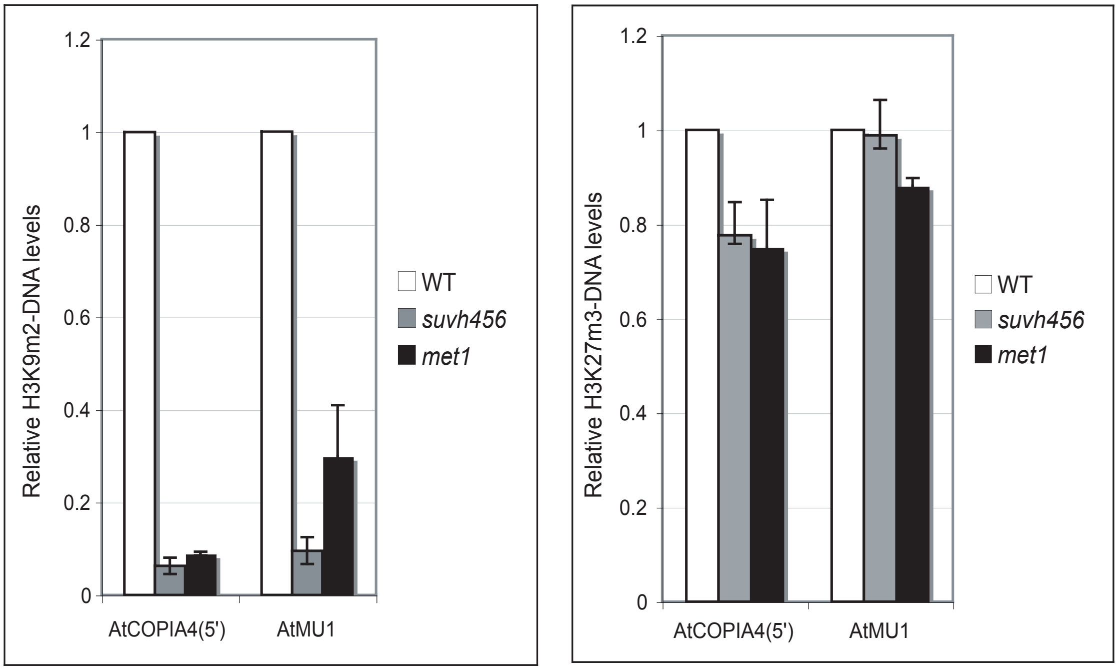

Supplemental Figure 7. Analysis of H3K9m2 and H3K27m3 marks at two transposons by ChIP followed by real-time PCR. Data were normalized to the input DNA and to an internal control (actin gene).
