# Supplementary material for: Loss of the DNA Methyltransferase MET1 Induces H3K9 Hypermethylation at PcG Target Genes and Redistribution of H3K27 Trimethylation to Transposons in Arabidopsis thaliana
Source: PLoS Genet. 2012 Nov 29;8(11):e1003062. doi: 10.1371/journal.pgen.1003062 (PMC3510029; doi:10.1371/journal.pgen.1003062)
Supplement: Figure S8 — Chromosomal distributions of H3K27m3 (left) and H3K9m2 (right) regions. Left and right panels show the normalized levels of H3K27m3 and H3K9m2 respectively in a sliding 100 kilobase windows. The black arrow indicates the centromeric regions. The blue arrow indicates the heterochromatic knob on chromosome 4. (PDF) [file pgen.1003062.s008.pdf]

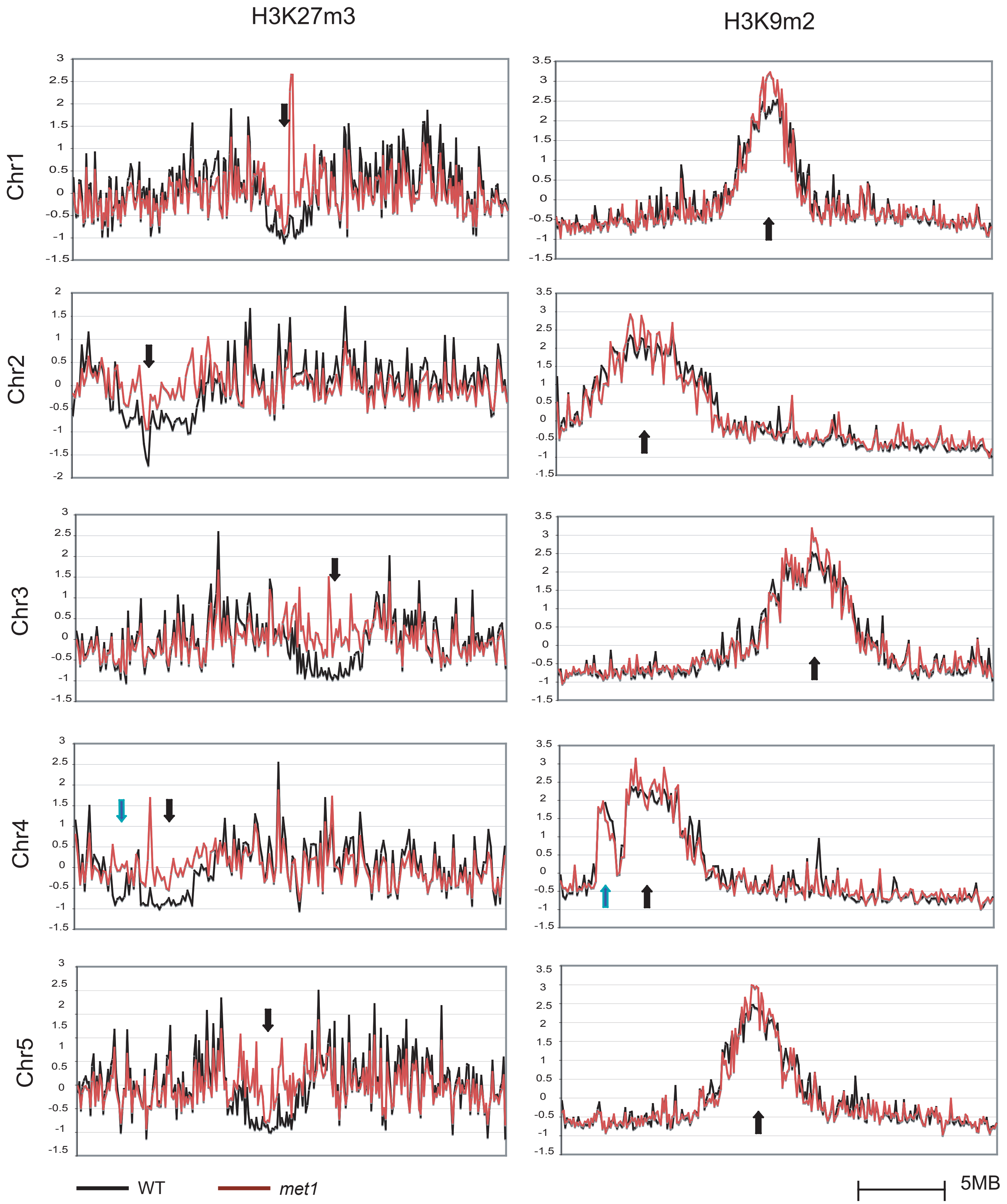

Supplemental Figure 9. Chromosomal distributions of H3K27m3 (left) and H3K9m2 (right). Left and right panels show the normalized levels of H3K27m3 and H3K9m2 respectively in a sliding 100 kilobase windows. The black arrow indicates the centromeric region. The blue arrow indicates the heterochromatic knob on chromosome 4.
