# Supplementary material for: Loss of the DNA Methyltransferase MET1 Induces H3K9 Hypermethylation at PcG Target Genes and Redistribution of H3K27 Trimethylation to Transposons in Arabidopsis thaliana
Source: PLoS Genet. 2012 Nov 29;8(11):e1003062. doi: 10.1371/journal.pgen.1003062 (PMC3510029; doi:10.1371/journal.pgen.1003062)
Supplement: Figure S9 — Supplementary information on ChIP-chip validation by ChIP-qPCR. Data shown in Figure 4C and Figure 5C are normalized to input, with actin shown as a separate control so that ChIP efficiencies can be visualized. (PDF) [file pgen.1003062.s009.pdf]

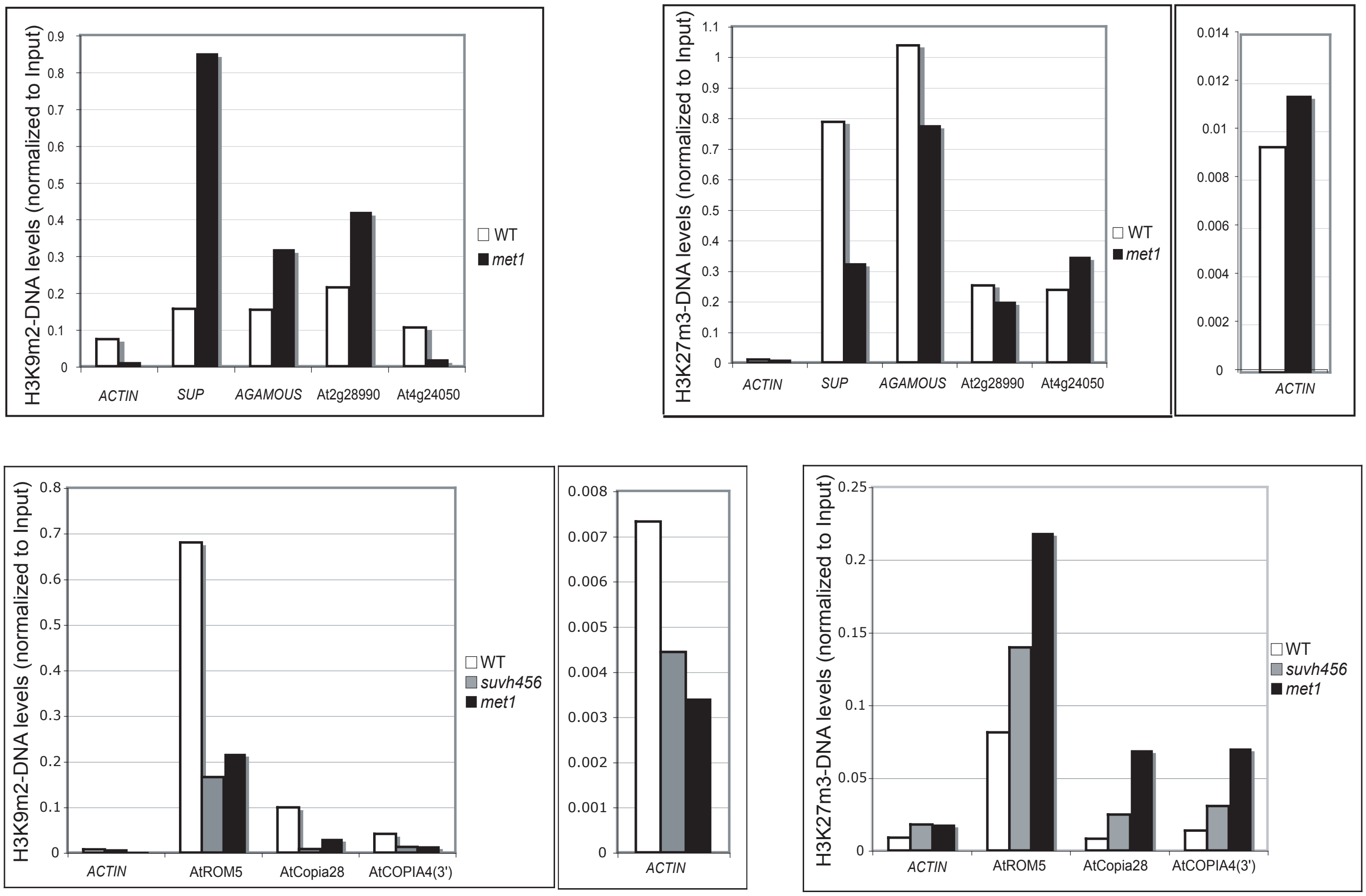

Supplemental Figure 9. Supplementary information on ChIP-chip validation by ChIP-qPCR. Data shown in Figures 4C (top panel) and 5C (bottom panel) are normalized to input, with actin shown as a separate control so that ChIP efficiencies can be visualized. One of the two experiments averaged in Figure 3C and 4C is shown here as a representative graph.
